# *Nigella sativa* and *Trigonella foenum-graecum* Supplemented Chapatis Safely Improve HbA1c, Body Weight, Waist Circumference, Blood Lipids, and Fatty Liver in Overweight and Diabetic Subjects: A Twelve-Week Safety and Efficacy Study

**DOI:** 10.1089/jmf.2020.0075

**Published:** 2020-09-02

**Authors:** Amit S. Rao, Shyamala Hegde, Linda M. Pacioretty, Jan DeBenedetto, John G. Babish

**Affiliations:** ^1^ Supreem Pharmaceuticals Mysore Pvt. Ltd., KIADB Industrial Area, Mysore, India.; ^2^ Bio Nexus Ltd., Brooktondale, New York, USA.

**Keywords:** *hepatic steatosis*, Nigella sativa, *obesity*, Trigonella foenum-graecum, *type 2 diabetes*

## Abstract

In 2019, ∼ 463 million people globally had diabetes mellitus (DM), with China (25.1%), India (16.6%), and the United States (6.69%) representing nearly 50% of that total. The primary objectives of this exploratory study were to assess the safety and potential efficacy of *Nigella sativa* and fenugreek seed supplemented chapatis in overweight (OW) and type 2 DM subjects. Forty subjects (15/OW; 9/DM; 16/DM/OW) consumed two chapatis twice a day 6 days/week for a daily dose of 5.45 g of an *N. sativa*/fenugreek combination over 12 weeks with no changes in lifestyle or medications. Anthropometric, glycemic, and vascular variables were recorded at baseline and after 6 and 12 weeks. Glycated hemoglobin (HbA1c), plasma lipids, and complete metabolic profile were measured at baseline and termination. Compliance, estimated during twice-daily individual delivery of precooked chapatis, was 100%, with no significant adverse effects. At termination, body weights, body mass index, waist and hip circumferences, index of central obesity, HbA1c, fasting blood glucose, 2-h postprandial blood glucose, estimated average glucose over 12 weeks, total cholesterol (TC), non-high density lipoprotein (HDL) cholesterol, very low density lipoprotein (VLDL), and triglycerides (TG) were decreased (*P* < .05) over all subjects. Subjects with HbA1c ≥7.0 exhibited greater improvements in all glycemic variables than HbA1c <7.0 subjects. In addition, the decrease in HbA1c was positively correlated with decreases in (1) hepatic enzymes alkaline phosphatase (*r* = 0.301, *P* = .0067) and aspartate transaminase (*r* = 0.277, *P* = .0129), (2) systolic blood pressure (*r* = 0.388, *P* = .0004), and (3) number of diagnostic metabolic syndrome criteria exhibited per subject (*r* = 0.391, *P* = .0005), cardiovascular risk score (CRS) (*r* = 0.281, *P* = .0115), and hepatic steatosis index (HSI) (*r* = 0.223, *P* = .0467). Atherogenic and diabetogenic indexes TC/HDL, low density lipoprotein/HDL, VLDL/HDL, and TG/HDL were all decreased (*P* < .05). Among all subjects, improvement (*P* < .05) was seen in CRS (−10.7%), fatty liver index (−19.8%), lipid accumulation product (−13.8%), and HSI (−7.53%). *N. sativa*/fenugreek supplemented chapatis present a safe and seamless dietary modification to address cardiometabolic risk.

## Introduction

It has been estimated that in 2019, ∼ 463 million people globally were diabetic (∼90% type 2 diabetes mellitus [DM]), with China (25.1%), India (16.6%), and the United States (6.69%) representing nearly 50% of that total. In addition, increases in the worldwide prevalence of diabetes of 25% and 51% are projected over the next 10 and 25 years, respectively.^1^ In India alone, the prevalence of type 2 diabetes (T2D), as well as prediabetes,^i^ has been determined to be ∼12.1% and 14.0%, respectively, with no differences between genders.^2^ Prediabetes is generally asymptomatic, and studies suggest that it goes largely undiagnosed until the development of T2D or its complications.^2^ Furthermore, meta-analyses have revealed that T2D and its precursor prediabetes are a likely consequence of the “NAFLD pathway”, in which nonalcoholic fatty liver disease^ii^ (NAFLD) establishes multiple sites of metabolic dysregulation (multiple-hit pathogenesis) that become strong determinants for progression to prediabetes, metabolic syndrome (MetS), and T2D.^3^

Along this generally asymptomatic NAFLD pathway to the development of T2D, the number and types of developing comorbidities differ along geographical and ethnic lines. Macrovascular complications, such as coronary heart disease, peripheral vascular disease, and cerebrovascular disease are the leading cause of morbidity and mortality in the United States.^4^ In India patients with T2D exhibited a two-fold risk of coronary artery disease-related deaths compared with white, T2D Europeans.^5^

These disease demographics, which account for the second-largest market by sales in the pharmaceutical industry after cancer,^6^ have spurred the development of additional antidiabetic therapeutics with newly discovered metabolic targets, such as glucagon-like peptide-1 receptor agonists.^7^ When therapeutic intervention for T2D or associated liver disease is warranted, however, metformin remains the recommended drug of choice.^8^ Moreover, drugs alone are neither economically nor clinically sustainable. In addition to the obvious financial burden of a pharmaceutical solution, factors such as a monotherapy failure rate approaching 50% over 5 years,^9^ and low patient compliance due to the complexity of treatment for developing comorbidities,^10^ impose significant impediments to the management of T2D through current therapeutic guidelines. In support of a multiple-hit pathophysiology, studies have now indicated that early antidiabetic drug failure is related to increased hepatic transaminase levels within the normal range.^11^ Such small increases in aspartate aminotransferase (AST) and alanine transaminase (ALT) may be early indicators of the NAFLD pathway activation toward the development of T2D and reflective of a multitude of simultaneous, macro- and micro-metabolic alterations occurring within the patient. Moreover, its asymptomatic nature precludes therapeutic intervention.

Because there are no approved or effective therapeutics, the recommended treatment of NAFLD and nonalcoholic steatohepatitis (NASH) remains lifestyle modifications focusing on diet, weight loss, and increasing physical activity.^12^ These same lifestyle interventions have been shown to be more effective than therapeutics alone for glycemic control in T2D.^13^ While changing lifestyle behaviors can be difficult to achieve, providing staple foods with functional medical properties through phytoceutical supplementation can assist in seamlessly modifying the diet to support metabolic goals. The addition of phytoceuticals to staple foods affords the advantage of providing a benefit with little change in dietary habits. In choosing a phytoceutical supplement with the appropriate organoleptic appeal, indigenous spices and flavorings are obvious candidates. Two well-known spices in India, *Nigella sativa* (kalonji or black jeera)^14^ and *Trigonella foenum-graecum* (methi),^15^ have been widely used in a variety of foods as a spice, flavoring agent, and traditional remedy.

*N. sativa* seeds, alone or in combination with honey, have been used for centuries to treat various human conditions, including (1) obesity, (2) hyperglycemia, (3) elevated plasma lipids, (4) cardiovascular disorders, (5) systemic inflammation, (6) infectious diseases, and (7) wounds.^14^ Similarly, *T. foenum-graecum* (fenugreek) has been cited in ancient texts, as well as Ayurveda and traditional Chinese medicine. Clinically reported uses of fenugreek, which largely overlap with *N. sativa*, include (1) weight loss, (2) hyperglycemia, (3) elevated plasma lipids, (4) systemic inflammation, (5) microbial infections, (6) increasing postpartum milk secretion, and (7) general health improvement.^16^ Fenugreek is also used to enhance the sensory quality of foods as a stabilizer, emulsifier, and adhesive.^15^

The recognition of the wide-ranging, potential health benefits of *N. sativa* and its high margin of safety has encouraged scientists to incorporate *N. sativa* into food matrices such as bread containing ground seeds^17,18^ and cookies formulated with fixed seed oil or thymoquinone.^19–21^ Fenugreek bread has also been suggested as a functional food for the management of T2D.^22^ Indeed, fenugreek powder, formulated in buns, flatbreads, and whole wheat bread, has been shown to decrease the glycemic response in healthy,^23^ overweight (OW), and obese,^24^ as well as diet-controlled T2D, subjects.^25^ While encapsulated combinations of *N. sativa* and fenugreek have been clinically tested in T2D subjects,^26–29^ no food combinations of these phytoceuticals have been clinically evaluated. We hypothesized that an *N. sativa*/fenugreek combination in a food that was consumed daily in the diet could act on multiple complementary targets in the NAFLD pathway, prediabetes, and T2D. For our phytoceutical carrier, we chose chapatis. This wheat-based, traditional Indian flatbread is an established part of the Indian daily diet.^30^ Furthermore, it has been reported that obese individuals eat significantly larger quantities of chapatis than normal-weight persons.^31^

The primary objectives of this exploratory study were to assess the safety and potential efficacy of powdered *N. sativa* and fenugreek seed supplementation of a staple food in OW and T2D subjects. Based on the reported efficacy of these two spices, we hypothesized that the fortified chapatis would normalize glycemic variables, as well as variables associated with hepatic and cardiovascular disease risk.

## Materials and Methods

### Study design

This study was an exploratory, interventional, single-arm 12-week trial conducted at the JSS Ayurveda Medical Hospital in Mysore, India. Prior approval of the clinical protocol was provided by the Ethics Committee of the JSS Ayurveda Medical Hospital. Wheat flour chapatis, fortified to provide, respectively, 4.7 and 0.75 g powdered *N. sativa* and fenugreek seed/day with the consumption of four chapatis (Table 1), were supplied 6 days/week for 12 weeks to OW, T2D (DM), or DM and OW subjects (DM/OW). Participants were instructed not to modify their diet, physical activity, lifestyle habits, current prescription drugs, or dietary supplements.

**Table 1.** Macronutrient and Supplement Content of Wheat Flour, Supplemented Wheat Flour, and Daily Chapati Consumption

Macronutrient composition	Wheat flour (g/100 g)	Supplemented flour*^a^*(g/100 g)	Chapatis*^b^*(g/day)
Calories (kcal)	323	320	300
Moisture	11.0	10.8	76.4
Carbohydrate	61.0	57.5	53.9
Total ash	1.43	1.76	1.65
Crude protein	14.4	14.7	13.8
Total fat	2.32	3.43	3.22
Saturated fatty acids	0.570	0.770	0.722
Monounsaturated fatty acids	0.380	0.630	0.591
Polyunsaturated fatty acids	1.37	2.03	1.90
Trans fatty acids	ND	ND	ND
Dietary fiber	9.83	10.9	10.26
Insoluble dietary fiber	8.87	10.0	9.41
Soluble dietary fiber	0.960	0.904	0.848
Seed powder supplement			
Whole *Nigella* powder		2.50	2.35
Defatted *Nigella* seed powder		2.50	2.35
Ground fenugreek seed powder		0.800	0.750

^a^ Commercial whole wheat flour (Pillsbury chakki fresh atta 100% atta, 0% maida).

^b^ One kilogram of the supplemented wheat flour was mixed with 700–750 mL water to form dough; 45–50 g of the dough were grilled resulting in finished chapatis weighing 38–45 g.

ND, none detected with limit of quantitation = 0.10%.

### Subject selection

**FIG. 1.**
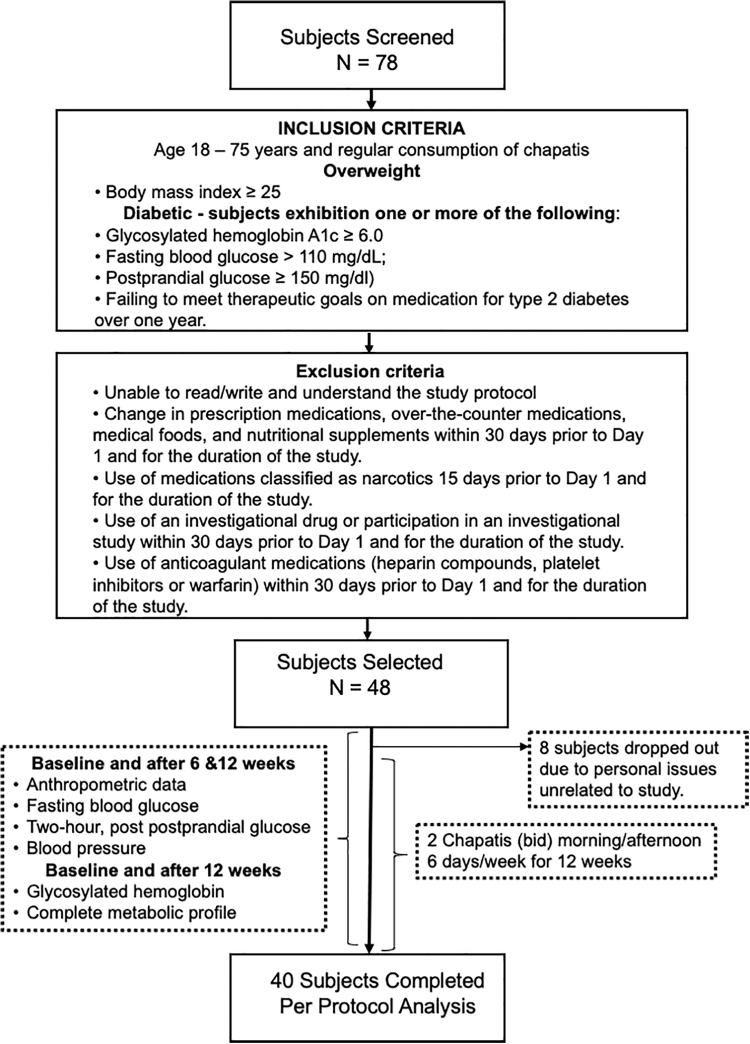
Schematic representation of clinical trial. Seventy-eight subjects were evaluated, and 48 were selected based upon entrance and exclusion criteria. Subjects between the ages of 18–75 with a body mass index >25 (OW) or type 2 (DM) subjects with two or more of the following: HbA1c ≥6.0, fasting blood glucose >110 mg/dL, postprandial glucose ≥150 mg/dL, or on medication for type 2 diabetes over 1 year without adequate blood sugar control were selected to participate. Those individuals with both type 2 DM and overweight were classified as DM/OW. Participants were not required to modify their diet, physical activity, lifestyle habits, current prescription drugs, or dietary supplements. Of the 48 selected to participate, 8 dropped out within the first weeks due to personal issues unrelated to the study. Forty subjects completed the study with 100% compliance. DM, diabetic; OW, overweight; HbA1c, glycated hemoglobin.

Subjects for this study were recruited in Mysore, India beginning September 25, 2018, with completion of the trial on April 26, 2019. Recruitment notices, describing the trial, were posted in relevant departments of the JSS Ayurveda Medical Hospital in Mysore and the local newspaper to advertise the study. Forty-eight of 78 subjects screened were selected based upon entrance, exclusion criteria, and regular consumption of chapatis, as well as willingness to participate 6 days/week for 12 weeks (Fig. 1). Briefly, individuals between the ages of 18–75 with a body mass index (BMI) >25 (OW) or T2D (DM) subjects with one or more of the following: glycated hemoglobin (HbA1c) ≥6.0, fasting blood glucose (FBG) >110 mg/dL, postprandial glucose (PPG) ≥150 mg/dL, or on medication for T2D over 1 year without adequate blood sugar control were selected to participate. Those individuals meeting both T2D and OW criteria were classified as DM/OW. Concurrent medications of 2 months or longer were not discontinued for the trial.

### Investigational product—*N. sativa* and fenugreek fortified chapatis

Commercial whole wheat flour (Pillsbury chakki fresh atta 100% atta, 0% maida) was formulated to contain 5% *N. sativa* (kalonji) and 0.8% fenugreek (methi) ground seed. The identity of the commercial *N. sativa* and fenugreek seeds was confirmed at Supreem Pharma (Mysore, India). One-half of the *N. sativa* seed powder (2.5% of the formulated Atta flour) was a defatted seed powder produced by supercritical carbon dioxide extraction (Table 1).^32^ As a result of flour supplementation, the fat content increased by 49%, representing 66% and 48% increases in monounsaturated fatty acids and polyunsaturated fatty acids, respectively. Chapatis were prepared twice daily at Supreem Pharma by adding 700–750 mL of water to 1 kg of supplemented wheat flour to form the chapatis dough; balls of 45–50 g; and the dough were pressed and grilled using Emami Healthy and Tasty refined sunflower oil (Emami Agrotech Ltd., Kolkata, India) resulting in finished chapatis weighting 38–45 g (*N. s*ativa/fenugreek*/*chapatis [NFC]). Two precooked NFC were delivered to each subject twice per day, Monday through Saturday. With each delivery, subjects were questioned concerning compliance, including changes in diets or physical activity, taste, and tolerance of the NFC.

### Clinical measurements

Clinical measurements and frequency are described in Tables 2 and 3, Supplementary Data S1 outlined in Figure 1. Anthropometric variables were measured at all clinical visits by a single physician of the JSS Ayurveda Medical Hospital assigned to monitor the trial. Subjects were requested to remove any outdoor clothes before measurements, breathe normally, and stand with feet fairly close together (about 12–15 cm apart) with weight equally distributed on each leg. For measuring of body weight (BW), a scale with accuracy 0.1 kg was used. Waist circumference (WC) was measured at the midpoint between the lowest rib and the top of the hip bone (iliac crest) with a flexible plastic measuring tape. Blood pressure (BP) was measured using a standard sphygmomanometer after resting for 10 min. The measurement was taken from a bare left arm in a quiet room.

**Table 2.** Baseline Anthropometric and Metabolic Characteristics of the Three Subgroups

Variable	OW (*N* = 15)	DM (*N* = 9)	DM/OW (*N* = 16)
Anthropometric^#^			
Gender M/F (%M)	5/10 (33.3%)	9/0 (100%)	5/11 (68.7%)
Male age (year/range)	37.0^a^ (32–42)	54.4^b^ (42–63)	54.2^b^ (41–72)
Female age (year/range)	34.4^a^ (23–45)	—	45.9^b^ (32–65)
Height (cm)	162^a^ ± 1.71	173^b^ ± 2.82	158^a^ ± 2.62
Weight (kg)	74.1^a^ ± 1.98	69.4^a^ ± 1.88	74.5^a^ ± 3.22
BMI (kg/m^2^)	28.3^b^ ± 0.657	23.3^a^ ± 0.439	29.6^b^ ± 0.776
Waist (cm)	92.1^a^ ± 1.92	90.4^a^ ± 1.72	97.3^b^ ± 2.36
Hip (cm)	108^b^ ± 1.60	97.9^a^ ± 0.920	106^b^ ± 2.55
Waist/hip	0.857^a^ ± 0.0206	0.924^b^ ± 0.0147	0.917^b^ ± 0.0191
Index of central obesity	0.569^a^ ± 0.00995	0.525^a^ ± 0.0116	0.615^b^ ± 0.0129
Glycemic^#^			
HbA1c ≥7.0 (% of subjects in group)	0/15 (0.0%)^a^	7/9 (77.8%)^b^	12/16 (75%)^b^
HbA1c (%)	5.29^a^ ± 0.0661	7.92^b^ ± 0.398	7.97^b^ ± 0.392
FBG (mg/dL)	92.9^a^ ± 1.99	137^b^ ± 15.1	164^b^ ± 11.3
PPBG (mg/dL)	129^a^ ± 4.30	228^b^ ± 24.7	232^b^ ± 14.9
eAG (mg/dL)	105^a^ ± 1.90	181^b^ ± 11.4	182^b^ ± 11.2
Vascular^#^			
SBP (mmHg)	120^a^ ± 2.05	123^a^ ± 2.89	126^a^ ± 2.88
DBP (mmHg)	76.9^a^ ± 1.36	80.7^a^ ± 3.56	80.9^a^ ± 2.26
MAP (mmHg)	91.3^a^ ± 1.28	94.9^a^ ± 3.08	95.9^a^ ± 2.35
PP (mmHg)	43.2^a^ ± 2.09	42.7^a^ ± 2.77	45.0^a^ ± 1.70
Lipidic^#^			
TC (mg/dL)	214^a^ ± 7.41	197^a^ ± 8.83	196^a^ ± 11.0
TC-HDL (mg/dL)	174^a^ ± 7.25	157^a^ ± 9.03	157^a^ ± 10.7
LDL (mg/dL)	137^a^ ± 7.13	127^a^ ± 8.47	126^a^ ± 9.45
VLDL (mg/dL)	32.3^a^ ± 1.88	30.9^a^ ± 2.95	27.8^a^ ± 2.12
HDL (mg/dL)	39.9^a^ ± 0.888	39.7^a^ ± 0.289	38.5^a^ ± 0.585
TG (mg/dL)	163^a^ ± 9.39	156^a^ ± 14.7	139^a^ ± 11.1
Lipidic ratios^#^			
TC/HDL	5.38^a^ ± 0.194	4.98^a^ ± 0.249	5.07^a^ ± 0.255
LDL/HDL	3.43^a^ ± 0.172	3.20^a^ ± 0.227	3.25^a^ ± 0.221
VLDL/HDL	0.815^a^ ± 0.0498	0.782^a^ ± 0.0782	0.719^a^ ± 0.0506
TG/HDL	4.21^a^ ± 0.297	3.69^a^ ± 0.285	3.53^a^ ± 0.323
Hepatic^#^			
GGT (IU/L)	16.5^a^ ± 1.07	23.1^b^ ± 1.97	15.7^a^ ± 1.93
AST (IU/L)	28.8^a^ ± 1.92	41.4^b^ ± 5.83	30.0^a^ ± 2.57
ALT (IU/L)	35.7^ab^ ± 2.64	44.3^b^ ± 5.58	31.6^a^ ± 2.47
AST/ALT	0.836^a^ ± 0.0564	0.925^a^ ± 0.0450	0.976^a^ ± 0.0695
ALP (IU/L)	148^a^ ± 8.45	175^a^ ± 17.9	167^a^ ± 13.5
Bilirubin (mg/dL)	0.653^a^ ± 0.0477	0.611^a^ ± 0.0807	0.644^a^ ± 0.0545
Direct bilirubin (mg/dL)	0.220^a^ ± 0.0200	0.222^a^ ± 0.0465	0.213^a^ ± 0.0256
Total protein (mg/dL)	6.59^a^ ± 0.108	6.88^a^ ± 0.134	6.76^a^ ± 0.102
Albumin (g/dL)	4.27^a^ ± 0.114	4.39^a^ ± 0.170	4.36^a^ ± 0.104
Globulin (g/dL)	2.32^a^ ± 0.104	2.43^a^ ± 0.124	2.41^a^ ± 0.0566
A/G	1.93^a^ ± 0.160	1.86^a^ ± 0.163	1.83^a^ ± 0.0676
Renal^#^			
BUN (mg/dL)	24.5^a^ ± 1.29	23.0^a^ ± 1.74	26.8^a^ ± 1.56
Creatinine (mg/dL)	0.880^a^ ± 0.0327	0.989^a^ ± 0.0484	0.994^a^ ± 0.0295
BUN/creatinine	28.6^a^ ± 2.22	23.2^a^ ± 1.17	27.3^a^ ± 1.91
Thyroid^#^			
T3 (ng/mL)	1.16^a^ ± 0.0475	1.17^a^ ± 0.0512	1.17^a^ ± 0.0616
T4 (mg/dL)	7.83^a^ ± 0.483	8.00^a^ ± 0.299	7.90^a^ ± 0.655
TSH (mg/dL)	3.19^a^ ± 0.439	2.77^a^ ± 0.306	3.48^a^ ± 0.584
Metabolic syndrome^#^			
Number of diagnostic criteria met	2.20^a^ ± 0.296	2.33^a^ ± 0.167	3.31^b^ ± 0.254
Cardiometabolic indices^##^			
Cardiovascular risk (%)	1.20^a^ (0.700–2.90)	11.9^b^ (4.20–23.1)	3.75^b^ (0.800–38.8)
Fatty liver index (score)	42.0^b^ (25.0–80.0)	34.0^a^ (24.0–45.0)	50.0^b^ (25.0–84.0)
Lipid accumulation product (score)	58.1^b^ (27.8–115)	41.2^a^ (29.2–57.6)	52.4^ab^ (31.7–98.2)
Hepatic steatosis index (score)	39.1^b^ (34.6–48.8)	33.5^a^ (32.2–36.8)	38.4^b^ (31.9–46.3)

^#^ Values are mean ± SEM; common letter superscripts indicate nonsignificant differences *P* > .05 determined by ANOVA.

^##^ Values are medians with parenthetic range; common letter superscripts indicate nonsignificant differences *P* > .05 determined by Kruskal–Wallis test.

ALP, alkaline phosphatase; ALT, alanine transaminase; AST, aspartate aminotransferase; BMI, body mass index; BUN, blood urea nitrogen; DBP, diastolic blood pressure; DM, diabetic; OW, overweight; eAG, estimated average glucose; FBG, fasting blood glucose; GGT, gamma-glutamyl transferase; HbA1c, glycated hemoglobin; HDL, high density lipoprotein; ICO, index of central obesity; LDL, low density lipoprotein; MAP, mean arterial pressure; OW, over weight; PP, pulse pressure; PPG, postprandial glucose; SBP, systolic blood pressure; T3, triiodothyronine; T4, thyroxine; TC, total cholesterol; TG, triglycerides; TSH, thyroid stimulating hormone; VLDL, very low density lipoprotein; SEM, standard error of mean; ANOVA, analysis of variance.

**Table 3.** Mean Baseline Cardiometabolic Profiles and Mean Percent Changes from Baseline After Six and Twelve Weeks

Variable	Baseline (*N* = 40)	Week 6 (% change)	P value	Week 12 (% change)	P value	P-trend^a^
Anthropometric^a^						
Weight (kg)	73.2 ± 1.55	−1.79 (−2.46 to −1.13)	<.0001	−2.12 (−3.13 to −1.10)	.0001	<.0001
BMI (kg/m^2^)	27.7 ± 0.560	−1.80 (−2.48 to −1.11)	<.0001	−2.33 (−3.68 to −0.982)	.0012	<.0001
Waist (cm)	93.8 ± 1.31	−2.20 (−3.12 to −1.27)	<.0001	−3.25 (−4.26 to −2.24)	<.0001	<.0001
Hip (cm)	105.0 ± 1.329	−1.39 (−2.15 to −0.639	.0006	−1.81 (−2.87 to −0.753)	.0013	<.0001
Waist/hip	0.896 ± 0.0121	−0.845 (−1.92 to 0.233)	.1208	−1.50 (−2.78 to −0.233)	.0234	.0081
Index of central obesity	0.577 ± 0.00867	−2.16 (−3.03 to −1.29)	<.0001	−3.25 (−4.21 to −2.29)	<.0001	<.0001
Glycemic^a^						
HbA1c (%)	6.95 ± 0.272	—	—	−5.65 (−8.59 to −2.85)	.0002	NC
FBG (mg/dL)	131 ± 7.49	−9.31 (−18.1 to −0.420)	.0405	−6.95 (−15.0 to 1.05)	.0866	.1034
PPBG (mg/dL)	193 ± 11.3	−7.46 (−16.4 to 1.52)	.1012	−8.39 (−16.2 to 0.663)	.0341	.0465
eAG (mg/dL)	153 ± 7.82	—	—	−7.39 (−11.1 to −3.71)	.0002	NC
Vascular^a^						
SBP (mmHg)	123 ± 1.55	−0.285 (−1.42 to 0.854)	.6149	0.992 (−1.33 to 3.32)	.3911	.3129
DBP (mmHg)	79.4 ± 1.31	−1.07 (−3.26 to 1.12)	.3285	−1.45 (−5.88 to 2.98)	.5131	.4239
MAP (mmHg)	94.0 ± 1.27	−0.734 (−2.17 to 0.704)	.3085	−0.381 (−3.35 to 2.59)	.7969	.7563
PP (mmHg)	43.8^a^ ± 1.19	1.14 (−5.87 to 8.15)	.7465	5.43 (−1.58 to 12.42)	.1275	.1275
Lipidic^a^						
TC (mg/dL)	203 ± 5.61	—	—	−7.88 (−12.4 to −3.33)	.0012	NC
TC-HDL (mg/dL)	163 ± 5.49	—	—	−9.39 (−15.9 to −3.77)	.0016	NC
LDL (mg/dL)	130 ± 4.96	—	—	−6.68 (−13.6 to 0.304)	.0603	NC
VLDL (mg/dL)	30.2 ± 1.29	—	—	−11.1 (−17.9 to −4.30)	.0020	NC
HDL (mg/dL)	39.3 ± 0.416	—	—	−1.91 (−4.1 to 0.270)	.0842	NC
TG (mg/dL)	152 ± 6.61	—	—	−9.21 (−14.9 to −3.51)	.0022	NC
Lipidic ratios^a^						
TC/HDL	5.16 ± 0.137	—	—	−5.85 (−10.5 to −1.15)	.0161	NC
LDL/HDL	3.31 ± 0.119	—	—	−4.23 (−11.1 to 2.61)	.2189	NC
VLDL/HDL	0.769 ± 0.0325	—	—	−9.44 (−16.9 to −1.94)	.0150	NC
TG/HDL	3.87 ± 0.166	—	—	−7.44 (−13.7 to −1.18)	.0211	NC
Hepatic^a^						
GGT (IU/L)	17.7 ± 0.849	—	—	−13.5 (−22.5 to −4.37)	.0048	NC
AST (mg/dL)	32.1 ± 1.93	—	—	−16.5 (−24.8 to −8.15)	.0003	NC
ALT (mg/dL)	36.0 ± 1.98	—	—	−17.8 (−26.1 to −9.42)	<.0001	NC
AST/ALT	0.912 ± 0.0369	—	—	2.47 (−5.19 to 10.1)	.6564	NC
ALP (mg/dL)	162 ± 7.45	—	—	−12.7 (−18.3 to −6.92)	<.0001	NC
Bilirubin (mg/dL)	0.640 ± 0.0354	—	—	−16.1 (−27.8 to −4.17)	.0093	NC
Direct bilirubin (mg/dL)	0.218 ± 0.0160	—	—	−6.88 (−25.6 to 11.8)	.4602	NC
Protein (mg/dL)	6.73 ± 0.0659	—	—	−1.45 (−3.06 to 0.171)	.0781	NC
Albumin (g/dL)	4.34 ± 0.0693	—	—	−3.06 (−5.09 to −1.01)	.0044	NC
Globulin (g/dL)	2.38 ± 0.0520	—	—	1.89 (−2.72 to 6.51)	.4123	NC
Albumin/globulin	1.87 ± 0.0737	—	—	−5.88 (−13.1 to 1.35)	.1078	NC
Renal^a^						
Urea (mg/dL)	25.1 ± 0.892	—	—	−5.78 (−12.2 to 0.637)	.6564	NC
Creatinine (mg/dL)	0.948 ± 0.0218	—	—	−1.05 (−5.81 to 3.70)	.6564	NC
Urea/creatinine	26.9 ± 1.18	—	—	−5.95 (−13.3 to 1.36)	.6564	NC
Thyroid^a^						
T3 (ng/mL)	1.17 ± 0.0318	—	—	−0.206 (−5.60 to 5.18)	.9389	NC
T4 (mg/dL)	7.89 ± 0.319	—	—	1.16 (−3.05 to 5.36)	.5812	NC
TSH (mg/dL)	3.21 ± 0.291	—	—	−2.07 (−17.0 to 12.9)	.7803	NC

^a^ Baseline values are mean ± SEM; differences from baseline were determined by paired-*t* or repeated measures ANOVA and are represented as mean percent difference from baseline with parenthetic 95% confidence interval; *P*-trend represents result of analysis for nonzero slope over 12 weeks.

NC, not calculated.

Mean arterial pressure (MAP) was computed as (SBP +2 × DBP)/3 and pulse pressure (PP) = (SBP − DBP). Estimated average glucose (eAG) over 12 weeks was computed using the formula: eAG (mg/dL) = [(28.7 × HbA1c) −46.7].^33^ Analysis of whole blood and serum samples was conducted by the JSS Ayurveda Medical Hospital diagnostic laboratory. Reference ranges for the interpretation of complete metabolic profile were based on historical hospital records and as published.^34^

### Cardiometabolic algorithms

In addition to waist, hip, and waist/hip measurements, the index of central obesity (ICO) was computed as a parameter of central obesity using the formula: WC (cm)/Height (cm).^35^ The National Cholesterol Education Program Adult Treatment Panel-III guidelines were used to determine the number of MetS diagnostic criteria exhibited by each subject.^36^ The atherosclerotic cardiovascular disease algorithm (http://static.heart.org/riskcalc/app/index.html#!/baseline-risk) was used to estimate the risk of stroke or heart disease over 10 years (cardiovascular risk score [CRS]).^37^ The fatty liver index (FLI) was calculated from serum triglycerides (TG), BMI, gamma-glutamyl transferase (GGT), and WC according to the equation: FLI = (e^0.953×loge (triglycerides) +0.139^ ^×BMI +0.718×loge (GGT) +0.053×WC −15.745^)/(1 + e^0.953×loge (TG) +0.139×BMI +0.718×loge (GGT) +0.053×WC −15.745^) × 100.^38^ The lipid accumulation product (LAP) was computed, respectively, for men and women as: LAP_M_ = (WC [cm] −65) × TG (mmol/L), LAP_W_ = (WC [cm] −58) × TG (mmol/L).^38^ The formula: HSI = 8 × ALT/AST + BMI (+2 if T2D yes, +2 if female) was used to compute the hepatic steatosis index (HSI).^39^

### Ethics

The clinical protocol was approved by the Ethics Committee of the JSS Ayurveda Medical Hospital, Mysore. This study was conducted based on good clinical practice International Conference on Harmonisation guidance and the ethical principles of the Declaration of Helsinki. Before enrollment, every participant received complete instructions concerning the protocol and the objectives of the study in nontechnical terms, and they then executed a written, informed consent document. A personal copy of the executed, informed consent document was provided to each subject.

### Statistical methods

An earlier, single-arm study analyzed by one of the authors (J.G.B.) was used to estimate sample size.^40^ In this previous trial, a −4.1% difference (*P* < .01) from a median baseline value for HbA1c of 5.8% was detected using the paired *t*-test. From this effect size and standard deviation of the difference, a group size of 35 was estimated for hemoglobin A1c (HbA1c) and glycemic variables, with the probability of a type I error at 5% and type II error at 20%. A goal of 40 subjects, therefore, was established to account for possible attrition. A per protocol analysis was conducted with subjects completing the trial with compliance set at 100%.

Clinical response variables and the number of MetS criteria met were normally distributed and analyzed using paired *t*-test or one-way, repeated measures analysis of variance (ANOVA). The *post hoc* analysis of the HbA1c ≥7.0 subgroup versus HbA1c <7.0 subgroup analysis was conducted using a two-way, fixed effects ANOVA model. Cardiometabolic indices, including CRS, FLI, LAP, and HSI, and their percent change from baseline were not normally distributed and analyzed using the nonparametric Wilcoxon matched-pairs signed-rank test. Tabulated baseline values are presented as mean ± standard error of mean for normally distributed data and medians with parenthetic ranges for nonparametric results. Ninety-five percent confidence intervals of the relative differences were computed for both means and medians. Individual subject data and abridged ANOVA tables for all variables and indexes are presented in Supplementary Figures S1–S7. Pearson's linear correlation coefficient *r* was used to assess the relationship between HgA1c and other variables. StatMate and GraphPad Software (San Diego, CA, USA) were used, respectively, for power and statistical analysis. Using two-tailed tests, the probability of a type I error was set at the nominal 5% level for all variables.

## Results

### Subject engagement and compliance

After initial, in-person screening interviews, which included medical records of 78 subjects, 48 were selected to participate based on inclusion criteria (Fig. 1). Eight subjects withdrew from the study in the first weeks due to personal reasons unrelated to the trial. Due to the twice-daily contact with the subjects over 12 weeks, 40 completed the trial with 100% compliance (*i.e.*, no noteworthy changes in diet or physical activity and consumption of NFC two times/day, 6 days/week).

### Baseline description of the subjects

Overall, 47.5% (19 M/21 F) of the subjects were male with a mean age of 49.8 years and a range of 32–72 years. The mean age and range for females were 40.4 years (23–65). Fifteen subjects were classified as OW, 9 as DM, and 16 as DM/OW with males comprising 5/15 (33%), 9/9 (100%), and 5/16 (68.7%) of each subgroup, respectively. At 37.0 years, OW males were younger than DM males (54.4 years; *P* = .0049) and OW/DM males (54.2 years; *P* = .0131). Similarly, OW females were younger than DM/OW females 34.4 versus 45.9 and *P* = .0068 (Table 2).

Eight (88.9%) and 11 (68.8%) of the nine DM and 16 DM/OW subjects, respectively, were taking prescription drugs, ayurvedic medications, or supplements. Median years on medication were 5.0 and 2.8 years for DM and DM/OW, respectively. In the DM subgroup, metformin (one subject), metformin with glimepiride (four subjects), and insulin (one subject) were the prescribed drugs, while combinations of antidiabetic ayurvedic medications and bitter melon (one subject), as well as bitter melon alone (one subject), were taken by two subjects. Eight (50.0%) DM/OW subjects were receiving oral hyperglycemic agents (OHA), including metformin (one subject), combinations of metformin with glimepiride, sitagliptin, or teneligliptin (five subjects), glimepiride (one subject), or glimepiride plus insulin (one subject). One of these eight was also prescribed insulin in addition to the metformin combinations. One subject was being treated with the antihypertensive agent metoprolol in addition to a metformin/glimepiride combination. Three DM/OW subjects (18.8%) were taking ayurvedic medications, one with a bitter melon supplement. A preliminary statistical analysis of HbA1c, FBG, eAG, and postprandial blood glucose (PPBG) at baseline, week 6, and week 12 in these subjects versus DM subjects that had not received OHA or taken supplements (*N* = 6) indicated no differences between these subgroups. Therefore, both OHA-medicated and nonmedicated diabetics were grouped for statistical analyses.

Adherence to protocol eligibility requirements is reflected in the baseline subgrouping descriptions (Table 2). While BWs were similar over subgroups, BMI was lower in DM than OW (*P* = .0003) or DM/OW (*P* < .0001) as DM were taller. WC and ICO were highest in the DM/OW subgroup. The percent of subjects with HbA1c ≥7.0 and %HbA1c was similar in the DM (7/9; mean HbA1c = 7.92%) and DM/OW (12/16; mean HbA1c = 7.97%) subgroups and greater than the OW (0/15; mean HbA1c = 5.29%). Similarly, FBG, eAG, and PPG were equal in DM and DM/OW and both elevated with respect to OW.

BPs, lipid profiles, and ratios, as well as kidney and thyroid function, did not differ among subgroups. At baseline, DM exhibited generally higher GGT, AST, ALT, and alkaline phosphatase (ALP) than OW or DM/OW, and mean AST (41.4 IU/L) and ALP (175 IU/L) exceeded the normal reference ranges of 40 and 147 IU/L, respectively. All other hepatic biomarkers were in the normal reference ranges and did not differ among subgroups.

Overall, being DM/OW put subjects at greatest risk of a diagnosis of MetS as 75% (95% confidence interval [CI] = 50.5–89.8%) of DM/OW exhibited ≥3 diagnostic criteria for MetS, while only 46.7% (24.8–69.9%) and 33.3% (12.1–64.6%) OW and DM, respectively, were diagnostic for MetS. Similarly, the mean number of diagnostic criteria met was greatest (*P* < .05) in the DM/OW subgroup 3.32 versus 2.20 and 2.33 for OW and DM, respectively (Table 2). The risk of a cardiovascular event over the next 10 years (CRS) was higher in the DM (11.9%) and DM/OW (3.75%) than in the OW (1.20%) subjects (*P* < .05 for both). FLI, LAP, and HSI, indices of hepatic fat accumulation, were elevated in the OW and DM/OW subgroups relative to the DM subjects (*P* < .05), possibly related to a lower BMI and WC in the DM subjects and reflecting differences in underlying metabolic dysfunction.

### Tolerability

Volunteers were given two chapatis b.i.d. (twice a day), as a regular diet, without restricting their normal food habits and no other special instruction in terms of exercise or workouts. NFC were well received, and their taste was rated “good” to “excellent” overall. Subjects routinely commented on feeling lighter and clothes fitting better during the study. Four subjects reported relief of chronic constipation, while five subjects initially recounted instances of transient gastric upset with four chapatis per day and were switched to three chapatis per day with no further issues.

### Anthropometric measurements

All anthropometric variables were positively modified at the completion of 12 weeks and exhibited a trend to increasing improvement (*P* < .0001) with time on the study (Table 3 and Supplementary Fig. S1). Specifically, mean BW was reduced −1.55 kg (95% CI = −2.29 to −0.808 kg; *P* = .0001), BMI −0.645 kg/m^2^ (−1.02 to −0.272 kg/m^2^; *P* = .0012), WC −3.05 cm (−4.00 to −2.10 cm; *P* < .0001), hip circumference (HC) −1.90 cm (−3.01 to −0.781 cm; *P* = .0013), waist/hip −1.50% (−2.78% to −0.214%; *P* = .0234), and ICO −3.25% (−4.21% to −2.29%; *P* < .0001). There were no differences in the relative changes in anthropometric measurements among criteria subgroups (data not shown).

### Glycemic responses

After 12 weeks of NFC consumption, mean absolute HbA1c change from baseline over all subjects was −0.393% (−0.587% to −0.198%; *P* = .0002) representing a relative HbA1c decrease of −5.65% (−8.58% to −2.85%). Concordant relative decreases were seen in FBG and PPBG of −9.31% (−18.1% to −0.420%; *P* = .0405) and −7.46% (−16.4% to 1.52%; *P* = .1012), respectively, at 6 weeks and −6.95% (−15.0% to 1.05%; *P* = .0866) and −8.39% (−16.2% to −0.663%; *P* = .0341) at 12 weeks. Moreover, PPBG decreases from baseline through 6 and 12 weeks exhibited a linear trend for improvement (*P* = .0465). eAG, which reflects the average blood glucose concentration over the previous 12 weeks, decreased −7.39% (−11.1% to −3.71%; *P* = .0002) from 153 to 142 mg/dL over all subjects (Table 3 and Supplementary Fig. S2).

**FIG. 2.**
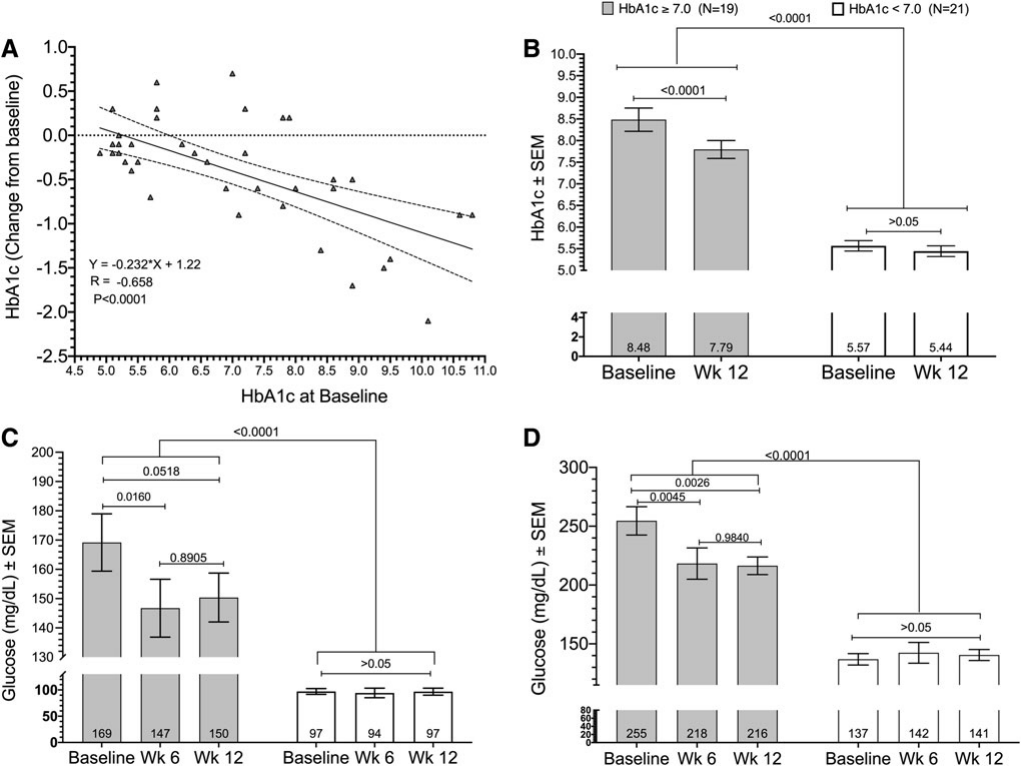
**(A)** Linear regression of absolute HbA1c changes over 12 weeks of consuming *Nigella sativa*/fenugreek supplemented chapatis 6 days per week as a function of baseline HbA1c. This association was linear with *r* = −0.658 (95% CI −0.804 to −0.436; *P* < .0001). *Dotted lines* represent 95% confidence belts. **(B)** Absolute HbA1c changes (±SEM) over 12 weeks for the HbA1c ≥7.0 and HbA1c <7.0 *post hoc* subgroups at baseline and 12 weeks. Mean absolute HbA1c decrease was −0.689 (−0.942 to −0.436; *P* < .001; *N* = 19) for HbA1c ≥7.0 subgroup versus no change (*P* > .05) for the HbA1c <7.0 subgroup. **(C)** FBG mg/dL (±SEM) over 12 weeks for the HbA1c ≥7.0 and HbA1c <7.0 *post hoc* subgroups, respectively, at baseline, 6 weeks, and 12 weeks. FBG fell −22.4 mg/dL (−3.51 to −41.3; *P* = .0160) and −18.8 mg/dL (−37.7 to 0.118; *P* = .0518) at 6 and 12 weeks, respectively, with no difference between changes at weeks 6 and 12 (*P* = .891). **(D)** PPBG mg/dL (±SEM) for the HbA1c ≥7.0 and HbA1c <7.0 *post hoc* subgroups at baseline, 6 weeks, and 12 weeks. PPBG fell −36.3 mg/dL (−62.8 to −9.82; *P* = .0045) and −38.2 mg/dL (−64.7 to −11.7; *P* = .0026), respectively, with no difference between changes at weeks 6 and 12 (*P* = .984). There were no differences from baseline in the HbA1c <7.0 subgroup for any of the glycemic variables at 6 or 12 weeks **(B**–**D)**. Analyses were conducted using a two-way, fixed effects ANOVA model. ANOVA, analysis of variance; CI, confidence interval; FBG, fasting blood glucose; PPBG, postprandial blood glucose; SEM, standard error of mean.

Among all variables, glycemic responses exhibited the most pronounced differences between the criteria subgroups. As a *post hoc* subgroup, DM subjects exhibited higher baseline HbA1c, FBG, PPBG, and eAG versus nondiabetics (Table 2). As expected, the relationships between HbA1c with FBG and PPBG were linear with (*r* = 0.839; *P* < .0001; *N* = 80) and (*r* = 0.870; *P* < .0001; *N* = 80) from baseline through week 12, respectively (data not shown). Moreover, the absolute change in HbA1c was linearly correlated to baseline HbA1c with *r* = −0.658 (*P* < .0001; *N* = 40) with a decrease of −0.232 in HbA1c for each unit increase in baseline HbA1c level (Fig. 2A). Therefore, *post hoc* subgroups of baseline HbA1c ≥7.0 (range 7.00–10.8; *N* = 19) and baseline HbA1c <7.0 (range 4.90–6.00; *N* = 21) were created to assess changes in HbA1c, FBG, and PPBG over 12 weeks as a function of DM severity (Fig. 2B–D, respectively).

After 12 weeks, absolute HbA1c fell −0.689 (−0.942 to −0.436; *P* < .0001) relative to baseline (Fig. 2B) in the HbA1c ≥7.0 subgroup. FBG fell −22.4 mg/dL (−3.51 to −41.3; *P* = .0160) and −18.8 mg/dL (−37.7 to 0.118; *P* = .0518) at 6 and 12 weeks, respectively (Fig. 2C). Over the same time periods, PPBG fell −36.3 mg/dL (−62.8 to −9.82; *P* = .0045) and −38.2 mg/dL (−64.7 to −11.7; *P* = .0026). There were no differences from baseline in the HbA1c <7.0 subgroup for any of the glycemic variables at 6 or 12 weeks (Fig. 2B–D).

### Blood pressure

Consumption of NFC exhibited no treatment effects on any of the four measures of vascular pressure. Variations in systolic blood pressure (SBP) by subject during the study did not result in categorical changes as recently defined (Optimal, Normal, High normal, or Grade 1 hypertension).^41^ In addition, no treatment effects were noted for MAP or PP, and a majority of subjects exhibited normal MAP (38/40) and PP (39/40) from baseline through week 12 (Table 3 and Supplementary Fig. S3).

### Lipids

After 12 weeks, total cholesterol, non-high density lipoprotein (HDL) cholesterol, very low density lipoprotein (VLDL), and TG were decreased, respectively, −7.88% (−12.4% to −3.33%; *P* = .0012), −9.39% (−15.0% to −3.77%; *P* = .0016), −11.1% (−17.9% to −4.30%; *P* = .0020), and −9.21% (−14.9% to −3.51%; *P* = .0022) by NFC (Table 3 and Supplementary Fig. S4). Neither low density lipoprotein (LDL) nor HDL was changed from baseline *P* = .0603 and *P* = .0842, respectively. No differences were seen in the relative changes in lipid biomarkers when adjusted for DM severity (data not shown).

### Lipidic ratios

After 12 weeks of consuming NFC, decreases from mean baseline values (*N* = 40) were noted for TC/HDL, VLDL/HDL, and TG/HDL, respectively, −5.85% (−10.5% to −1.15%; *P* = .016), −9.44% (−16.9% to −1.94%; *P* = .015), and −7.44% (−13.7% to −1.18%; *P* = .021). LDL/HDL was not changed from baseline (Table 3 and Supplementary Fig. S5). No differences were seen in the responses of the lipidic ratios among criteria subgroups (data not shown).

### Hepatic function

Positive modifying effects were observed in hepatic biomarkers GGT −13.5%; (−22.5% to −4.37%; *P* = .0048), AST −16.5% (−24.8% to −8.15%; *P* = .0003), ALT −17.8% (−26.1% to −9.42%; *P* < .0001), ALP −12.7% (−18.3% to −6.92%; *P* < .0001), and bilirubin −16.1% (−27.8% to −4.17%; *P* = .0093). Mean ALP dropped from 162 IU/L, above the normal reference range of 147 IU, to 141 IU/L at the completion of 12 weeks (Table 3 and Supplementary Fig. S6). Relative responses for all hepatic biomarkers were similar among criteria subgroups (data not shown).

### Renal and thyroid assessment

No adverse effects on renal or thyroid function were seen from consuming the NFC over 12 weeks (Table 3 and Supplementary Fig. S7). Average blood urea nitrogen (BUN), creatinine, and BUN/creatinine were unchanged from baseline values with 95% CIs of percent change, respectively, (−12.2% to 0.637%; *P* = .0761), (−5.81% to 3.70%; *P* = .6564), and (−13.3% to 1.36%; *P* = .1080), respectively.

Similarly, mean triiodothyronine (T3), thyroxine (T4), and thyroid stimulating hormone did not change during the study with 95% CIs of percent change, respectively, (−5.60% to 5.18%; *P* = .939), (−3.05% to 5.35%; *P* = .581), and (−17.0% to 12.9%; *P* = .780) (Table 3 and Supplementary Fig. S7). No differences were seen in relative renal or thyroid changes among the criteria subgroups (data not shown).

### Cardiometabolic indices

**FIG. 3.**
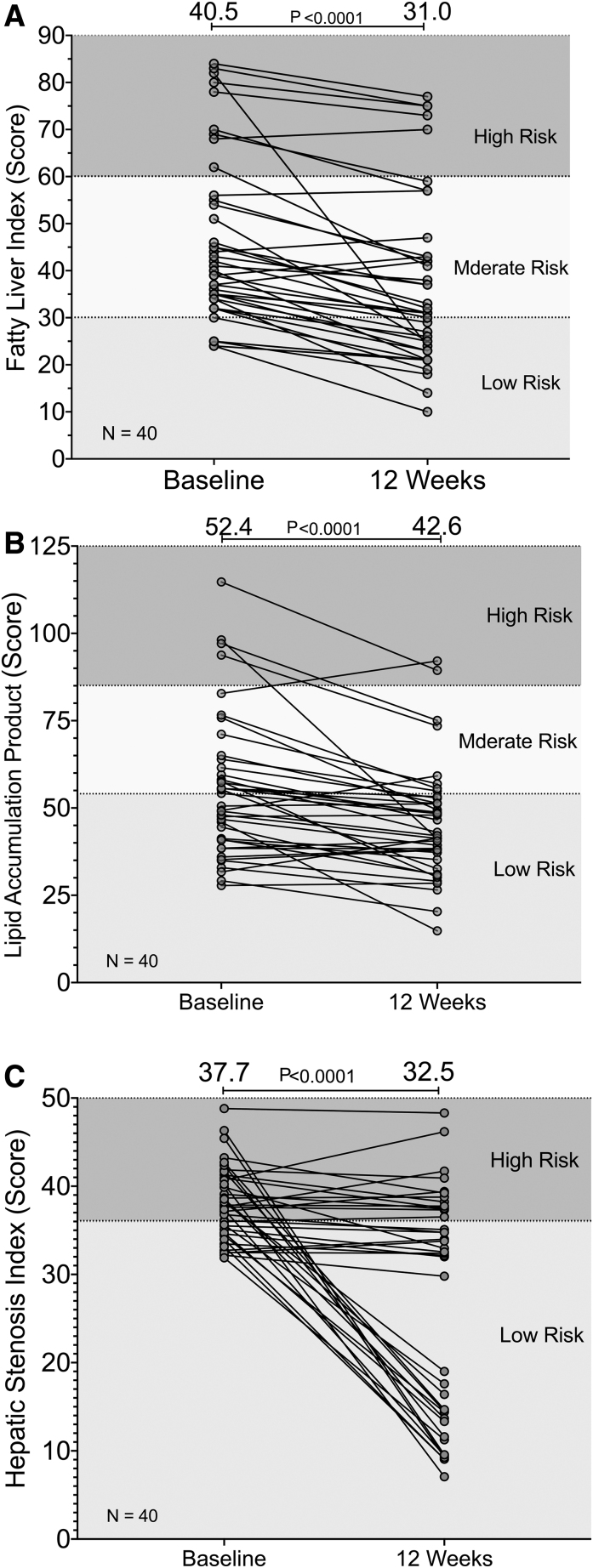
Individual changes in **(A)** fatty liver index with median change −19.8% (95% CI = −27.2 to −14.8; *P* < .0001), **(B)** lipid accumulation product with median change −13.8% (−24.6 to −10.6; *P* < .0001), and **(C)** hepatic stenosis index with median change −7.53% (−53.1 to −2.76; *P* < .0001) for all subjects from baseline through 12 weeks of consuming *N. sativa*/fenugreek supplemented chapatis 6 days per week. *Shaded areas* represent low risk, moderate risk, and high risk. Indexes were computed as described in “Materials and Methods,” section and data were analyzed using the nonparametric Wilcoxon matched-pairs signed rank test (*N* = 40).

Along with an −11.2% (−22.4% to 0.0135%; *P* = .0503) decrease in mean number of diagnostic criteria per subject, the CRS, whose variables partially overlap with MetS criteria, exhibited a median −10.7% decrease (−14.3% to −3.57%; *P* = .0021) over all subjects after 12 weeks (data not shown). The measures of hepatic fat accumulation, FLI, LAP, and HSI, all decreased significantly with median decreases of −19.8% (−27.2% to −14.8%), −13.8% (−24.6% to −10.6%), and −7.53% (−53.1% to −2.76%) with *P* < .0001 for all, respectively (Fig. 3).

## Discussion

Our initial flour formulations of 5% ground *N. sativa* and 1.0% fenugreek seeds resulted in a bitter taste with negative organoleptic ratings in informal taste tests (data not shown). The final formulation of 2.5% and 2.5% ground, defatted, and regular *N. sativa* seeds was derived from research demonstrating a hepatoprotective effect of *N. sativ*a defatted seed waste in a rodent model,^42^ and our work identified the *in vitro* lipolytic and AMPK activation activity of supercritical carbon dioxide, defatted *N. sati*va ground seeds.^32^ Simultaneously, the ground fenugreek seed concentration was reduced to 0.8% to further reduce bitterness. These changes resulted in a taste profile preferred over unfortified, whole wheat flour chapatis in a 20- to 50-year-old Indian demographic (pers. comm., Pune Focus Group, Saidapet, Kanchipuram, Tamil Nadu, India).

### Anthropometric

Anthropometric variables serve as basic components of validated algorithms for estimating the risk of cardiovascular and hepatic diseases.^35–38,43,44^ In our study, all anthropometric variables were positively affected at 6 and 12 weeks with a linear trend for improvement. These results are similar to a study of T2D patients with BMI = 28.01 kg/m^2^, FBG >133, and HbA1c >7.0 and no dietary or lifestyle modifications, in which subjects received 1:1 combinations of encapsulated *N. sativa* and fenugreek at doses ranging from 1000 to 2000 mg/day with glibenclamide. After 3 months, the authors reported a mean BMI decrease of −2.46% (*P* < .001 treatment × time).^27^ This BMI decrease is equivalent to our −2.33% (95% CI = −3.68% to −0.982%). In a bread matrix, MetS patients consuming 3 g powdered *N. sativa* did not exhibit significant decreases in BW, BMI, WC, SBP, DBP, or FBG. With regard to the commercial weight loss product orlistat, our mean weight loss of −2.12% (95% CI = −3.13% to −1.10%) after 12 weeks compared favorably to the mean weight loss of −2.50% reported in an observational study in 20 Caucasian nondiabetic females with a mean BMI of 45.2 kg/m^2^ after 6 months on a low fat diet.^45^

### Glycemic response

In addition to the positive relationships between HbA1c with FBG and PPBG previously described, significant positive relationships with HbA1c were also found for (1) hepatic enzymes ALP (*r* = 0.301, *P* = .0067) and AST (*r* = 0.277, *P* = .0129); (2) vascular biomarkers SBP (*r* = 0.388, *P* = .0004) and MAP (*r* = 0.2695, *P* = .0156); and (3) cardiometabolic indexes, including MetS criteria met (*r* = 0.391, *P* = .0005), CRS (*r* = 0.281, *P* = .0115), and HSI (*r* = 0.223, *P* = .0467). Collectively, these positive associations of hepatic enzymes, HSI, vascular biomarkers, and cardiovascular risk with HbA1c appear to depict a series of concordant metabolic alterations along the NAFLD pathway from minor liver perturbations to prediabetes and finally T2D.

Zarvandi *et al.*^29^ reported a single-arm trial of a polyherbal formulation with severely T2D patients on antidiabetic and statin medications with a mean baseline HbA1c of 8.4%. Subjects received two 6.4 g packets per day over 40 days for a daily dose of 600 mg *Allium sativum*, 600 mg *Aloe vera*, 3.6 g *N. sativa*, 2 g *Plantago psyllium*, 5.0 g *T. foenum-graecum*, and 1000 mg *Silybum marianum* extract. The polyherbal formulation significantly decreased FBG −16 mg/dL from an initial value of 162 mg/dL versus our baseline difference of −22 mg/dL (−44 to −0.70; *P* = .0160) in our HbA1c ≥7.0 (mean HbA1c = 8.48) subgroup at 6 weeks from a baseline of 169 mg/dL (Fig. 2B). No effect was seen with the polyherbal formulation on BWs, BP, liver, kidney, or hematological values.

Memon *et al.*^26^ studied the effect of 1:1 combinations of encapsulated *N. sativa* and fenugreek seeds at doses ranging from 1000 to 2000 mg/day along with glibenclamide in 50 patients with mean baseline FBG of 200 mg/dL. After 3 months, FBG decreased −58 and −1.0 mg/dL (*P* = .003) in treated and controls, respectively. This result also compared favorably with the −44 mg/dL decrease (95% CI = −70 to −18; *P* = .0003; *N* = 9) exhibited in our HbA1c ≥8.5 *post hoc* subgroup with baseline FBG of 196 mg/dL. Further in this same subgroup, absolute HbA1c decreased −1.12% (−1.57% to −0.679%; *P* = .0004; *N* = 9) in the same time frame (data not shown).

Studies of *N. sativa* and fenugreek administered separately in bread matrices were generally negative. MetS patients consuming 3 g powdered *N. sativa* daily in 100 g bread for 2 months did not exhibit significant decreases in FBG.^17^ In the second study, fenugreek administered as a single serving of 56 g of bread containing 2.8 g fenugreek failed to significantly affect PPBG in eight diet-controlled diabetics, although a decrease in insulin secretion over 2 h versus placebo was reported.^25^

### Vascular biomarkers

NFC consumption did not affect (*P* > .05) on mean SBP, DBP, MAP, or PP. A lack of hypotensive effect in T2D subjects was also reported for a polyherbal encapsulated formulation of *N. sativa* and fenugreek that additionally contained *Allium sativum*, *Aloe vera*, *P. psyllium*, and *S. marianum* administered over 40 days.^29^ Using a food matrix consisting of 100 g bread containing 3 g *N. sativa* powder, Mohtashami^17^ reported no effect on SBP or DBP in 51 subjects with MetS, who consumed the formulated bread for 2 months.

### Lipidic variables

TC, non-HDL cholesterol, VLDL, and TG were decreased (*P* < .05) by NFC. To evaluate the degree to which our findings would impact cardiovascular health, we cite a recently published meta-analysis of 30 studies that were used for evaluating the U.S. Food and Drug Administration (FDA) heart health claim of soy protein.^46^ The authors of this study examined 42 treatment arms (*n* = 2913), with an average soy protein intake of 26.9 g. They report that soy protein consumption led to reductions in standard difference in mean LDL = −8.9 mg/dL (95% CI = −6.1 to −11.8), TC = −8.5 mg/dL (−5.4 to −11.3), and TG of −7.09 mg/dL (−0.354 to −14.0).^46^ These results are in agreement with our findings for the reduction of LDL, TC, and TG of −8.7, −16, and −14 mg/dL, respectively. Thus, NFC reductions in lipid biomarkers equaled or exceeded those produced by consuming 26.9 g soy protein/day, which supports the FDA approved claim for heart health. These authors conclude, “…small, highly significant reductions in total and LDL cholesterol, equivalent to ca. 6% LDL reduction, particularly when combined with other dietary measures, can make a useful contribution to blood cholesterol management.”

The combined effects of encapsulated *N. sativa* and fenugreek on serum TG, HDL, and creatinine levels were evaluated in T2D patients with FBG >133 and HbA1c >7.0.^28^ Using 1:1 combinations of 1000–2000 mg/day with glibenclamide the authors reported −1.17% (*P* = .075) and +3.98% (*P* = .013) changes in TG and HDL, respectively, in the treated group versus no changes in TG or HDL in the control group. The polyherbal formulation of Zarvandi *et al.*^29^ previously described reduced TC, LDL, and TG, respectively, −17%, −21%, and −18% with no change in HDL over 42 days. Furthermore, MetS patients consuming 3 g powdered *N. sativa* in 100 g bread daily for 2 months exhibited no change (*P* > .05) in TC, LDL, HDL, TG, or LDL/HDL.^18^

### Lipidic indexes

“Atherogenic and diabetogenic indexes” derived from lipoprotein ratios such as TC/HDL, LDL/HDL, VLDL/HDL, and TG/HDL have been validated as biomarkers of (1) cardiovascular disease, (2) arterial stiffness, and (3) insulin resistance in OW, normotensive, and DM subjects.^47–51^ The −5.85% (−10.5% to −1.15%; *P* = .0161) and −7.44% (−13.7% to −1.18%; *P* = .0211) decreases, respectively, in TC/HDL and TG/HDL reflected improved glycemic control by NFC in DM and DM/OW individuals, as well as decreased cardiovascular disease risk and heart failure risk.^50,52,53^ In this study TC/HDL and TG/HDL were positively correlated with improvements in markers of insulin resistance such as LAP (*r* = 0.478; and *r* = 0.669, respective; *P* < .0001 for both), FLI (*r* = 0.320, *P* = .0038; *r* = 0.304, *P* = .0061, respectively), and number of MetS defining criteria (*r* = 0.271, *P* = .0149; *r* = 0.262, *P* = .0190), while only TC/HDL was positively correlated with improvements in BMI (*r* = 0.232, *P* = .038).

### Hepatic and fatty liver indices

The hepatic transaminase ALT dropped an average of 6.4 U over 12 weeks. It has been reported that “higher liver transaminase levels are associated with oral hypoglycemic agent failure” and that the relative risk for oral antidiabetic therapeutic failure falls by 1.7 for every 5-U decrease in ALT levels.^11^ Based on this relationship, our results indicate a 2.2-fold decrease in relative risk of therapeutic failure in T2D subjects receiving oral hypoglycemic agents with the consumption of NFC.

Multicomponent algorithms, such as ICO, FLI, HSI, and LAP, help understand the global interactions of multiple metabolic variables in NAFLD, NASH, and MetS; they have also demonstrated usefulness in diagnosing and monitoring their progression to T2D.^54–56^ Moreover, in the Indian population, LAP is a better predictor of MetS compared to BMI and WC.^57^ In concert with the improvements in GGT, ALT, AST, and ALP, all indices of fatty liver and MetS were improved with a majority of subjects demonstrating both lower risk categorical improvements, as well as quantitative changes in FLI, HSI, and LAP (Fig. 3). These changes in liver indexes are also reflective of the decreases in CRS and the number of diagnostic criteria met for MetS noted in this study. Individually, both *N. sativa* and fenugreek have been used successfully to treat NAFLD with doses ranging from 2 g/day for *N. sativa* to 20–50 g/day for fenugreek.^58,59^ In a 2017 placebo-controlled trial, NAFLD patients receiving 1 g powdered *N. sativa* capsules b.i.d. for 12 weeks experienced dramatic improvements in hepatic transaminases with 57% of the treated patients exhibiting normal fatty liver grading on ultrasound after 12 weeks.^58^ These patients also experience an approximate 10% decrease in BMI.

### Safety

Consistent with published clinical data regarding *N. sativa* and fenugreek in combination or alone, the consumption of NFC had no adverse effect on hepatic, kidney, or thyroid functioning.^15,28,60,61^ While *in vitro* and animal studies describe the potential for an interaction of *N. sativa* or fenugreek phytoceuticals with drugs metabolized by hepatic CYP3A4,^62,63^ in our study one subject receiving metoprolol (a CYP3A4 substrate) therapy for hypertension maintained readings of 120, 80, 40, and 93 mmHg for SBP, DBP, PP, and MAP, respectively, from baseline through 12 weeks.

### Limitations

Our study has a few inherent limitations. Specifically, the study did not have a concurrent control group and relied on (1) measures of internal consistency, (2) validated algorithms incorporating multiple variables to confirm changes, and (3) comparisons with previously reported studies using comparable patient profiles with similar combinations of active ingredients or food matrices. Considering the anticipated demographics of potential consumers, the broad inclusion criteria, wide age range, and enrollment of OW and DM patients were considered advantages of the study design. Enrollment of DM patients nonresponsive to OHA was also considered an advantage in our exploratory study, but this inclusion also introduced limitations in interpretation. In particular, one limitation was an inability to assess whether results were due to *N. sativa*/fenugreek supplementation alone or additive/synergistic interactions with the OHA. Furthermore, participants were predominantly Asian Indians from a single location, and the generalizability of this clinical trial to other populations is unknown. Finally, the sustainability of the effects was not addressed with a follow-up observational period.

### Conclusion

Dietary modifications and exercise are first-line interventions for NAFLD and T2D. Fatty liver and prediabetes, however, are generally asymptomatic and they go largely undiagnosed until the development of NASH, T2D, or their complications. With such a long asymptomatic latency period, fundamentally changing one's diet can be difficult. Providing staple indigenous foods with functional medical properties through phytoceutical supplementation can assist in seamlessly modifying the diet to support metabolic goals with no change in dietary habits. Taken as a whole, these exploratory findings provide supporting data for examining *N. sativa*/fenugreek combinations on multiple sites of metabolic dysregulation in the NAFLD pathway, including hepatic steatosis, cardiometabolic risk, and glycemic control. Furthermore, these supplemented chapatis were well accepted, produced no adverse metabolic effects, and did not apparently interfere with common antidiabetic medications or supplements.

## Availability of Data and Materials

As subgroups from this study are currently being analyzed for further publications, raw data are not publicly available. Individual subject data are provided graphically with statistical tables for all time points as supplementary figures as described. Raw data will be furnished by the corresponding author upon reasonable request.

## Ethics Approval and Consent to Participate

Prior approval of the clinical protocol was provided by the Ethics Committee of the JSS Ayurveda Medical Hospital, Mysore, India. This study was conducted based on the ethical principles of the Declaration of Helsinki of 1975, as revised in 2008 and good clinical practice International Conference on Harmonisation guidance. Informed consent in writing was obtained from every subject before enrollment in the study, and a copy of the informed consent was provided to each.

## Supplementary Material

Supplemental data

Supplemental data

Supplemental data

Supplemental data

Supplemental data

Supplemental data

Supplemental data

Supplemental data

## Notes

^i^ “The World Health Organization has defined prediabetes as a state of intermediate hyperglycemia using two specific parameters, impaired fasting glucose (IFG) defined as fasting plasma glucose of 110–125 mg/dL and impaired glucose tolerance defined as 2 h plasma glucose of 140–200 mg/dL after ingestion of 75 g of oral glucose load or a combination of the two based on a 2 h oral glucose tolerance test. The American Diabetes Association includes HgA1c between 5.7% and 6.4% in addition to IGT of 140–200 mg/dL and uses a lower cutoff value for IFG between 100 and 125 mg/dL.”^3^

^ii^ The defining characteristic of NAFLD is excess fat stored in liver cells adversely modifying hepatic functioning.

## References

[1] Saeedi P, Petersohn I, Salpea P, *et al.*: Global and regional diabetes prevalence estimates for 2019 and projections for 2030 and 2045: Results from the International Diabetes Federation Diabetes Atlas, 9th edition. Diabetes Res Clin Pract 2019;157:107843 31518657 10.1016/j.diabres.2019.107843

[2] Ramachandran A, Snehalatha C, Kapur A, *et al.*: High prevalence of diabetes and impaired glucose tolerance in India: National Urban Diabetes Survey. Diabetologia 2001;44:1094–1101 11596662 10.1007/s001250100627

[3] Lonardo A, Ballestri S, Marchesini G, Angulo P, Loria P: Nonalcoholic fatty liver disease: A precursor of the metabolic syndrome. Dig Liver Dis 2015;47:181–190 25739820 10.1016/j.dld.2014.09.020

[4] Zheng Y, Ley SH, Hu FB: Global aetiology and epidemiology of type 2 diabetes mellitus and its complications. Nat Rev Endocrinol 2018;14:88–98 29219149 10.1038/nrendo.2017.151

[5] Forouhi NG, Sattar N, Tillin T, McKeigue PM, Chaturvedi N: Do known risk factors explain the higher coronary heart disease mortality in South Asian compared with European men? Prospective follow-up of the Southall and Brent studies, UK. Diabetologia 2006;49:2580–2588 16972045 10.1007/s00125-006-0393-2

[6] Kaur P, Mittal A, Nayak SK, Vyas M, Mishra V, Khatik GL: Current strategies and drug targets in the management of type 2 diabetes mellitus. Curr Drug Targets 2018;19:1738–1766 30051787 10.2174/1389450119666180727142902

[7] Patel H, Munir K, Sutherland S, Karanikas CA, Konig M: Efficacy of dulaglutide as a first injectable option for patients with type 2 diabetes: A post-hoc pooled analysis. Diabetes Ther 2019;10:2321–2330 31605302 10.1007/s13300-019-00709-9 PMC6848683

[8] Qaseem A, Barry MJ, Humphrey LL, Forciea MA: Oral pharmacologic treatment of type 2 diabetes mellitus: A clinical practice guideline update from the American College of Physicians. Ann Intern Med 2017;166:279–290 28055075 10.7326/M16-1860

[9] Jeon JY, Lee SJ, Lee S, *et al.*: Failure of monotherapy in clinical practice in patients with type 2 diabetes: The Korean National Diabetes Program. J Diabetes Investig 2018;9:1144–1152 10.1111/jdi.12801 PMC6123024 29328551

[10] Marcum ZA, Gellad WF: Medication adherence to multidrug regimens. Clin Geriatr Med 2012;28:287–300 22500544 10.1016/j.cger.2012.01.008 PMC3335752

[11] Irace C, Rossetti M, Carallo C, *et al.*: Transaminase levels in the upper normal range are associated with oral hypoglycemic drug therapy failure in patients with type 2 diabetes. Acta Diabetol 2012;49:193–197 21305325 10.1007/s00592-011-0261-5

[12] Hallsworth K, Adams LA: Lifestyle modification in NAFLD/NASH: Facts and figures. JHEP Rep 2019;1:468–479 32039399 10.1016/j.jhepr.2019.10.008 PMC7005657

[13] Garcia-Molina L, Lewis-Mikhael AM, Riquelme-Gallego B, Cano-Ibanez N, Oliveras-Lopez MJ, Bueno-Cavanillas A: Improving type 2 diabetes mellitus glycaemic control through lifestyle modification implementing diet intervention: A systematic review and meta-analysis. Eur J Nutr 2020;59:1313–1328 31781857 10.1007/s00394-019-02147-6

[14] Yimer EM, Tuem KB, Karim A, Ur-Rehman N, Anwar F: *Nigella sativa* L. (black cumin): A promising natural remedy for wide range of illnesses. Evid Based Complement Alternat Med 2019;2019:1528635 31214267 10.1155/2019/1528635 PMC6535880

[15] Wani SA, Kumar P: Fenugreek: A review on its nutraceutical properties and utilization in various food products. J Saudi Soc Agric Sci 2018;17:97–106

[16] Nagulapalli Venkata KC, Swaroop A, Bagchi D, Bishayee A: A small plant with big benefits: Fenugreek (*Trigonella foenum-graecum* Linn.) for disease prevention and health promotion. Mol Nutr Food Res 2017;61 [Epub ahead of print]; DOI: 10.1002/mnfr.2016.00950 28266134

[17] Mohtashami A: Effects of bread with *Nigella sativa* on blood glucose, blood pressure and anthropometric indices in patients with metabolic syndrome. Clin Nutr Res 2019;8:138–147 31089467 10.7762/cnr.2019.8.2.138 PMC6494753

[18] Mohtashami A, Mahaki B, Azadbakht L, Entezari MH: Effects of bread with *Nigella sativa* on lipid profiles, apolipoproteins and inflammatory factor in metabolic syndrome patients. Clin Nutr Res 2016;5:89–95 27152298 10.7762/cnr.2016.5.2.89 PMC4855045

[19] Sultan MT, Butt MS, Ahmad AN, Ahmad N, Amanullah M, Rizwana Batool R: Utilization of *Nigella sativa* L. essential oil to improve the nutritive quality and thymoquinone contents of baked products. Pak J Nutr 2012;11:910–915

[20] Sultan MT, Butt MS, Saeed F, Rizwana Batool R: *Nigella sativa* L. fixed oil supplementation improves nutritive quality, tocopherols and thymoquinone contents of cookies. Br Food J 2012;114:966–977

[21] Tauseef M, Butt M, Pasha I, Qayyum M, Saeed F, Ahmed W: Preparation and evaluation of dietetic cookies for vulnerable segments using black cumin fixed oil. Pak J Nutr 2011;10:451–456

[22] Hooda S, Jood S: Nutritional evaluation of wheat-fenugreek blends for product making. Plant Foods Hum Nutr 2004;59:149–154 15678723 10.1007/s11130-004-0024-3

[23] Robert SD, Ismail AA, Rosli WI: Reduction of postprandial blood glucose in healthy subjects by buns and flatbreads incorporated with fenugreek seed powder. Eur J Nutr 2016;55:2275–2280 26358163 10.1007/s00394-015-1037-4

[24] Robert SD, Ismail AA, Wan Rosli WI: *Trigonella foenum-graecum* seeds lowers postprandial blood glucose in overweight and obese individuals. J Nutr Metab 2014;2014:964873 25276421 10.1155/2014/964873 PMC4167814

[25] Losso JN, Holliday DL, Finley JW, *et al.*: Fenugreek bread: A treatment for diabetes mellitus. J Med Food 2009;12:1046–1049 19857068 10.1089/jmf.2008.0199

[26] Memon AR, Memon AR, Shah SS, Khand F, Shaikh IA, Khushk IA: Effect of combination of *Nigella sativa* and *Trigonella foenum-graecum* seeds with glibenclamide on blood sugar levels in type-2 diabetes mellitus patients. ISRA Med J 2010;2:46–51

[27] Memon AR, Memon AR, Shah SS, Khand F, Shaikh IA, Khushk IA: Effect of combination of *Nigella sativa* and *Trigonella foenum-graecum* seeds with glibenclamide on body mass index in type-2 diabetes mellitus patients. ISRA Med J 2010;2:10–15

[28] Memon AR, Shah SS, Memon AR, Naqvi ASHR: Effect of combination of *Nigella sativa* and *Trigonella foenum-graecum* with glibenclamide on serum triglyceride, HDL and creatinine levels in type-2 diabetes mellitus patients. Pak J Pharmacol 2012;29:1–6

[29] Zarvandi M, Rakhshandeh H, Abazari M, Shafiee-Nick R, Ghorbani A: Safety and efficacy of a polyherbal formulation for the management of dyslipidemia and hyperglycemia in patients with advanced-stage of type-2 diabetes. Biomed Pharmacother 2017;89:69–75 28214690 10.1016/j.biopha.2017.02.016

[30] Parimala KR, Sudha ML: Wheat-based traditional flat breads of India. Crit Rev Food Sci Nutr 2015;55:67–81 24915406 10.1080/10408398.2011.647121

[31] Satija A, Taylor FC, Khurana S, *et al.*: Differences in consumption of food items between obese and normal-weight people in India. Natl Med J India 2012;25:10–13 22680313

[32] Babish JG, Pacioretty LM, Debenedetto J: Compositions from *Nigella sativa*. U.S. Patent 9,180,155 B2. 1 1, 2015

[33] Nathan DM, Kuenen J, Borg R, *et al.*: Translating the A1C assay into estimated average glucose values. Diabetes Care 2008;31:1473–1478 18540046 10.2337/dc08-0545 PMC2742903

[34] Gowda S, Desai PB, Hull VV, Math AAK, Vernekar SN, Kulkarni SS: A review on laboratory liver function tests. Pan Afr Med J 2009;3:17–17 21532726 PMC2984286

[35] Blagojevic IP, Eror T, Pelivanovic J, Jelic S, Kotur-Stevuljevic J, Ignjatovic S: Women with polycystic ovary syndrome and risk of cardiovascular disease. J Med Biochem 2017;36:259–269 30568543 10.1515/jomb-2017-0020 PMC6287215

[36] Huang PL: A comprehensive definition for metabolic syndrome. Dis Model Mech 2009;2:231–237 19407331 10.1242/dmm.001180 PMC2675814

[37] Goff DC Jr., Lloyd-Jones DM, Bennett G, *et al.*: 2013 ACC/AHA guideline on the assessment of cardiovascular risk: A report of the American College of Cardiology/American Heart Association Task Force on Practice Guidelines. J Am Coll Cardiol 2014;63(25 Pt B):2935–2959 24239921 10.1016/j.jacc.2013.11.005 PMC4700825

[38] Cheng YL, Wang YJ, Lan KH, *et al.*: Fatty liver index and lipid accumulation product can predict metabolic syndrome in subjects without fatty liver disease. Gastroenterol Res Pract 2017;2017:9279836 28194177 10.1155/2017/9279836 PMC5282434

[39] Lee JH, Kim D, Kim HJ, *et al.*: Hepatic steatosis index: A simple screening tool reflecting nonalcoholic fatty liver disease. Dig Liver Dis 2010;42:503–508 19766548 10.1016/j.dld.2009.08.002

[40] Tripp ML, Dahlberg CJ, Eliason S, *et al.*: A low-glycemic, mediterranean diet and lifestyle modification program with targeted nutraceuticals reduces body weight, improves cardiometabolic variables and longevity biomarkers in overweight subjects: A 13-week observational trial. J Med Food 2019;22:479–489 31084538 10.1089/jmf.2018.0063

[41] Williams B, Mancia G, Spiering W, *et al.*: 2018 ESC/ESH guidelines for the management of arterial hypertension. Rev Esp Cardiol (Engl Ed) 2019;72:160 30704723 10.1016/j.rec.2018.12.004

[42] Michel CG, El-Dine NS, Fahmy SM, Ezzat SM, Nesseem DI, El-Alfy TS: Phytochemical and biological investigation of the extracts of *Nigella sativa* L. seed waste. Drug Test Anal 2011;3:245–254 21309000 10.1002/dta.225

[43] Lin MS, Lin TH, Guo SE, *et al.*: Waist-to-height ratio is a useful index for nonalcoholic fatty liver disease in children and adolescents: A secondary data analysis. BMC Public Health 2017;17:851 29084519 10.1186/s12889-017-4868-5 PMC5663116

[44] Castera L, Friedrich-Rust M, Loomba R: Noninvasive assessment of liver disease in patients with nonalcoholic fatty liver disease. Gastroenterology 2019;156:1264–1281.e4. 30660725 10.1053/j.gastro.2018.12.036 PMC7505052

[45] Valsamakis G, McTernan PG, Chetty R, *et al.*: Modest weight loss and reduction in waist circumference after medical treatment are associated with favorable changes in serum adipocytokines. Metabolism 2004;53:430–434 15045687 10.1016/j.metabol.2003.11.022

[46] Harland JI, Haffner TA: Systematic review, meta-analysis and regression of randomised controlled trials reporting an association between an intake of circa 25 g soya protein per day and blood cholesterol. Atherosclerosis 2008;200:13–27 18534601 10.1016/j.atherosclerosis.2008.04.006

[47] Millan J, Pinto X, Munoz A, *et al.*: Lipoprotein ratios: Physiological significance and clinical usefulness in cardiovascular prevention. Vasc Health Risk Manag 2009;5:757–765 19774217 PMC2747394

[48] Li G, Wu HK, Wu XW, *et al.*: Small dense low density lipoprotein-cholesterol and cholesterol ratios to predict arterial stiffness progression in normotensive subjects over a 5-year period. Lipids Health Dis 2018;17:27 29433526 10.1186/s12944-018-0671-2 PMC5810050

[49] Wen J, Zhong Y, Kuang C, Liao J, Chen Z, Yang Q: Lipoprotein ratios are better than conventional lipid parameters in predicting arterial stiffness in young men. J Clin Hypertens (Greenwich) 2017;19:771–776 28560757 10.1111/jch.13038 PMC8031038

[50] Zonszein J, Lombardero M, Ismail-Beigi F, *et al.*: Triglyceride high-density lipoprotein ratios predict glycemia-lowering in response to insulin sensitizing drugs in type 2 diabetes: A post hoc analysis of the BARI 2D. J Diabetes Res 2015;2015:129891 26106623 10.1155/2015/129891 PMC4461783

[51] Ray S, Talukdar A, Sonthalia N, *et al.*: Serum lipoprotein ratios as markers of insulin resistance: A study among non-diabetic acute coronary syndrome patients with impaired fasting glucose. Indian J Med Res 2015;141:62–67 25857496 10.4103/0971-5916.154504 PMC4405942

[52] Vega GL, Barlow CE, Grundy SM, Leonard D, DeFina LF: Triglyceride-to-high-density-lipoprotein-cholesterol ratio is an index of heart disease mortality and of incidence of type 2 diabetes mellitus in men. J Investig Med 2014;62:345–349 10.2310/JIM.0000000000000044 24402298

[53] Yunke Z, Guoping L, Zhenyue C: Triglyceride-to-HDL cholesterol ratio. Predictive value for CHD severity and new-onset heart failure. Herz 2014;39:105–110 23588603 10.1007/s00059-013-3788-0

[54] Zheng RD, Chen ZR, Chen JN, Lu YH, Chen J: Role of body mass index, waist-to-height and waist-to-hip ratio in prediction of nonalcoholic fatty liver disease. Gastroenterol Res Pract 2012;2012:362147 22701476 10.1155/2012/362147 PMC3369513

[55] Yadav D, Choi E, Ahn SV, *et al.*: Fatty liver index as a simple predictor of incident diabetes from the KoGES-ARIRANG study. Medicine (Baltimore) 2016;95:e4447 27495073 10.1097/MD.0000000000004447 PMC4979827

[56] Kahn HS: The “lipid accumulation product” performs better than the body mass index for recognizing cardiovascular risk: A population-based comparison. BMC Cardiovasc Disord 2005;5:26 16150143 10.1186/1471-2261-5-26 PMC1236917

[57] Ray L, Ravichandran K, Nanda SK: Comparison of lipid accumulation product index with body mass index and waist circumference as a predictor of metabolic syndrome in indian population. Metab Syndr Relat Disord 2018;16:240–245 29648916 10.1089/met.2017.0119

[58] Hussain M, Tunio AG, Akhtar L, Shaikh GS: Effects of *Nigella sativa* on various parameters in patients of non-alcoholic fatty liver disease. J Ayub Med Coll Abbottabad 2017;29:403–407 29076670

[59] Yarnell E, Abascal K: Herbal medicine and nonalcoholic fatty liver disease. Altern Complement Ther 2010;16:15–21

[60] Kandhare AD, Thakurdesai PA, Wangikar P, Bodhankar SL: A systematic literature review of fenugreek seed toxicity by using ToxRTool: Evidence from preclinical and clinical studies. Heliyon 2019;5:e01536 31049444 10.1016/j.heliyon.2019.e01536 PMC6482331

[61] Tavakkoli A, Mahdian V, Razavi BM, Hosseinzadeh H: Review on clinical trials of black seed (*Nigella sativa*) and its active constituent, thymoquinone. J Pharmacopuncture 2017;20:107–121 30087794 10.3831/KPI.2017.20.021 PMC5633670

[62] Ahmad A, Khan RM, Alkharfy KM, Raish M, Al-Jenoobi FI, Al-Mohizea AM: Effects of thymoquinone on the pharmacokinetics and pharmacodynamics of glibenclamide in a rat model. Nat Prod Commun 2015;10:1395–1398 26434126

[63] Al-Jenoobi FI, Alam MA, Alkharfy KM, *et al.*: Pharmacokinetic interaction studies of fenugreek with CYP3A substrates cyclosporine and carbamazepine. Eur J Drug Metab Pharmacokinet 2014;39:147–153 24022709 10.1007/s13318-013-0149-6

