# Supplementary material for: Nigella sativa and Trigonella foenum-graecum Supplemented Chapatis Safely Improve HbA1c, Body Weight, Waist Circumference, Blood Lipids, and Fatty Liver in Overweight and Diabetic Subjects: A Twelve-Week Safety and Efficacy Study
Source: J Med Food. 2020 Sep 2;23(9):905–19. doi: 10.1089/jmf.2020.0075 (PMC7478223; doi:10.1089/jmf.2020.0075)
Supplement: Supplemental data [file Supp_Data.pdf]

Supplementary Data S1

PROTOCOL SUMMARY

This study is a pilot, observational, open label 12-week trial conducted at the JSS Ayurveda Medical Hospital in Mysore, India. The objectives of the study are to evaluate the safety and efficacy of a wheat flour fortified with *Nigella sativa* and *Trigonella foenum-graecum* seed powder as functional food in obesity and type 2 diabetes mellitus. Forty overweight, obese, or type 2 diabetic subjects will be recruited from the JSS Ayurveda Medical Hospital in Mysore, India. Subjects will receive two whole wheat chapatis fortified with 5% ground *N. sativa* seed and 0.8% ground fenugreek seed twice daily for 6 days/week over 12 weeks (Table S1). Anthropometric variables, including body mass index (BMI), waist and hip circumference, blood pressure, as well as fasting blood glucose and a 2-h postprandial glucose, will be determined at baseline and completion of weeks 6 and 12. Glycated hemoglobin (HbA1c), lipidic biomarkers, and hepatic functioning variables along with renal and thyroid function will be measured at baseline and at the completion of week 12. Compliance and tolerance will be assessed daily during each delivery of formulated chapatis. Primary end points will be safety (*i.e.*, complete metabolic profile), tolerance, and glycemic variables. Secondary end points will include effects on anthropometric variables and blood lipids.

Table S1. Macronutrient and Supplement Content of Wheat Flour, Supplemented Wheat Flour, and Daily Chapati Consumption

Macronutrient composition	*Wheat flour (g/100 g)*	*Supplemented flour[a] (g/100 g)*	*Chapatis[b] (g/day)*
Calories (kcal)	323	320	300
Moisture	11.0	10.8	76.4
Carbohydrate	61.0	57.5	53.9
Total ash	1.43	1.76	1.65
Crude protein	14.4	14.7	13.8
Total fat	2.32	3.43	3.22
Saturated fatty acids	0.570	0.770	0.722
Monounsaturated fatty acids	0.380	0.630	0.591
Polyunsaturated fatty acids	1.37	2.03	1.90
Trans fatty acids	ND	ND	ND
Dietary fiber	9.83	10.9	10.26
Insoluble dietary fiber	8.87	10.0	9.41
Soluble dietary fiber	0.960	0.904	0.848
Seed powder supplement			
Whole *Nigella* powder		2.50	2.35
Defatted *Nigella* seed powder		2.50	2.35
Ground fenugreek seed powder		0.800	0.750

[a]Commercial whole wheat flour (Pillsbury chakki fresh atta 100% atta, 0% maida).

[b]One kilogram of the supplemented wheat flour was mixed with 700–750 mL water to form dough; 45–50 g of the dough were grilled resulting in finished chapatis weighing 38–45 g.

ND, none detected with limit of quantitation = 0.10%.

STUDY SUBJECTS

Subjects for this study will be recruited from the JSS Ayurveda Medical Hospital in Mysore, India. Flyers describing the details of the study and posted in relevant departments of the JSS Ayurveda Medical Hospital in Mysore, India will be used to recruit subjects.

Criteria for screening subjects for clinical study

Subjects between the ages of 18 and 75 with a BMI >25 or diabetic subjects with elevated HbA1c, fasting blood glucose, postprandial glucose, or on medication for type 2 diabetes over one year without adequate blood sugar control will be selected to participate.

Specific inclusion criteria

- Age 18–75 years

Overweight

- BMI ≥25

Diabetic—subjects exhibiting two or more of the following:

- Hemoglobin A1c ≥6.0
- Fasting blood glucose >110 mg/dL
- Postprandial glucose ≥150 mg/dL
- Failing to meet therapeutic goals on medication for type 2 diabetes over 1 year.

Exclusion criteria

- Unable to read/write and understand the study protocol
- Change in prescription medications, over-the-counter medications, medical foods, and nutritional supplements within 30 days prior to day 1 and for the duration of the study.
- Use of medications classified as narcotics 15 days prior to day 1 and for the duration of the study.
- Use of an investigational drug or participation in an investigational study within 30 days prior to day 1 and for the duration of the study.
- Use of anticoagulant medications (heparin compounds, platelet inhibitors, or warfarin) within 30 days prior to day 1 and for the duration of the study. Use of aspirin 81 or 325 mg once daily is permitted.

Number of subjects to be screened and statistical methodology

An earlier observational study conducted by this study supervisor was used to estimate sample size. In this previous study, a –4.1% difference ($P < .01$) from a median baseline

value for HbA1c of 5.8% was detected using the paired *t*-test. From this effect size and standard deviation of the difference, a group size of 35 was estimated for hemoglobin A1c (HbA1c) and glycemic variables with the probability of a type I error at 5% and type II error at 20%. Forty subjects, therefore, were recruited to account for possible attrition. An intent-to-treat analysis will be conducted using last visit carried forward for any missing values.

Normally distributed response variables will be analyzed using paired *t*-test or one-way repeated measures analysis of variance. Response variables not normally distributed will be analyzed using the nonparametric Wilcoxon Signed Rank Test. These statistical techniques allow the baseline value of each subject to serve as the control. Pearson's linear correlation coefficient *r* will be used to assess the relationship between variables. StatMate and GraphPad Software (San Diego, CA, USA) were used, respectively, for power and statistical analysis. Using two-tailed tests, the probability of a type I error will be set at the 5% level for all variables.

Study duration

Total length of participation in the study will be 12 weeks, excluding screening appointments.

Initial and follow-up investigations

0 week (Initial), 6 weeks, and 12 weeks.

- ''Initial data'' was taken at the beginning of the clinical trial (baseline).
- ''First follow-up'' is done at the end of 6 weeks (±2 days) after intervention.
- ''Second follow-up'' is done after completing the trial, that is, at the end of 3 months.

Anthropometric data collected at baseline and at the completion of six and twelve weeks

- Age—baseline only
- Gender—baseline only
- Height
- Weight
- BMI
- Waist and hip circumference measurement

Clinical parameters monitored at baseline and at the completion of six and twelve weeks

- Glycemic profile, including
  - HbA1c—baseline and week 12 only
  - Fasting blood glucose
  - 2-h, post postprandial glucose
- Systolic and diastolic blood pressure

Clinical parameters monitored at baseline and at the completion of twelve weeks

- Lipid profile
  - Cholesterol
  - Non high density lipoprotein (HDL) cholesterol
  - Low density lipoprotein cholesterol
  - Very low density lipoprotein cholesterol
  - HDL cholesterol
  - Triglycerides
- Liver function tests
  - Gamma-glutamyl transferase
  - Aspartate transaminase (AST)
  - Alanine transaminase (ALT)
  - AST/ALT
  - Alkaline phosphatase
  - Bilirubin
  - Direct bilirubin
  - Total protein
  - Albumin
  - Globulin
  - Albumin to globulin ratio
- Kidney function tests
  - Blood urea nitrogen
  - Creatinine
  - Blood urea nitrogen/creatinine ratio
- Thyroid function tests
  - T3—triiodothyronine
  - T4—thyroxine
  - TSH—thyroid stimulating hormone
- Abdomen and pelvic magnetic resonance imaging scanning will be performed for female subjects.

CONCOMITANT MEDICATIONS AND PROCEDURES

All concomitant medications taken during study participation will be recorded on the visit progress note. A prescription medication is defined as a medication that can be prescribed only by a properly authorized/licensed clinician. Medications to be reported are concomitant prescription medications, over-the-counter medications, medical foods, and nutritional supplements taken during the course of the study.

Chapati supplementation

All subjects will be receiving the test material consisting of two fortified chapatis twice per day for 6 days/week over 12 weeks.

Test material and dosing

The following materials will be used in the chapati test formulation

- Wheat flour—Pillsbury (0% maida)—obtained from commercial market
- *N. sativa* seeds—Black cumin seed (kalonji or black jeera)—Supreem Pharma
- Fenugreek seeds—Methi: *T. foenum-graecum*—obtained from commercial market
- Oil: Emami Healthy and Tasty refined sunflower oil
