# Supplementary material for: Nigella sativa and Trigonella foenum-graecum Supplemented Chapatis Safely Improve HbA1c, Body Weight, Waist Circumference, Blood Lipids, and Fatty Liver in Overweight and Diabetic Subjects: A Twelve-Week Safety and Efficacy Study
Source: J Med Food. 2020 Sep 2;23(9):905–19. doi: 10.1089/jmf.2020.0075 (PMC7478223; doi:10.1089/jmf.2020.0075)
Supplement: Supplemental data [file Supp_Fig1.pdf]

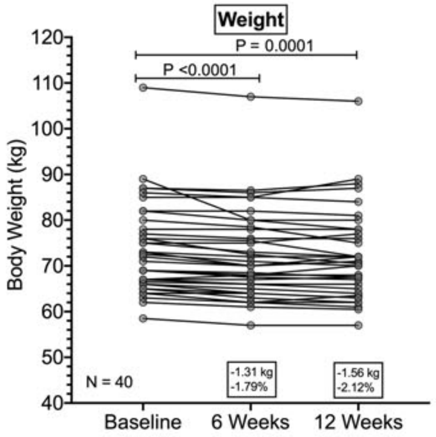

Uncorrected Fisher's LSD	Mean Diff.	95.00% CI of diff.	Significant?	Summary	Individual P Value
Baseline vs. 6 Weeks	1.313	0.8257 to 1.799	Yes	****	<0.0001
Baseline vs. 12 Weeks	1.550	0.8081 to 2.292	Yes	***	0.0001

	Baseline	6 Weeks	12 Weeks
Mean	73.21	71.90	71.66

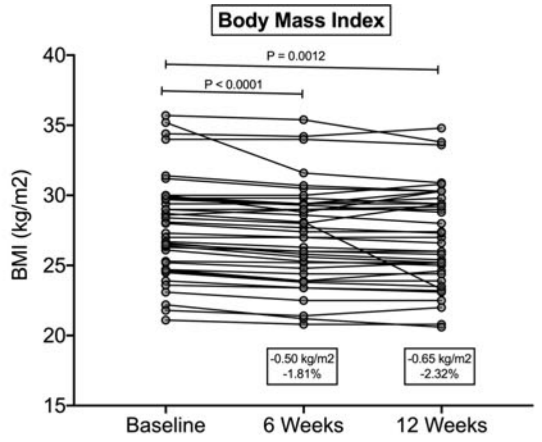

Uncorrected Fisher's LSD	Mean Diff.	95.00% CI of diff.	Significant?	Summary	Individual P Value
Baseline vs. 6 Weeks	0.4975	0.3071 to 0.6879	Yes	****	<0.0001
Baseline vs. 12 Weeks	0.6450	0.2723 to 1.018	Yes	**	0.0012

	Baseline	6 Weeks	12 Weeks
Mean	27.70	27.20	27.05

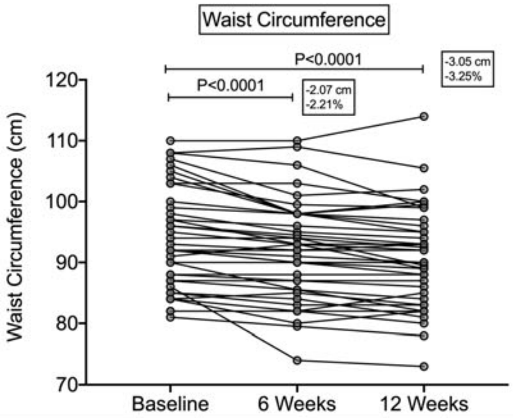

Uncorrected Fisher's LSD	Mean Diff.	95.00% CI of diff.	Significant?	Summary	Individual P Value
Baseline vs. 6 Weeks	2.063	1.195 to 2.930	Yes	****	<0.0001
Baseline vs. 12 Weeks	3.050	2.103 to 3.997	Yes	****	<0.0001

	Baseline	6 Weeks	12 Weeks
Mean	105.0	103.5	103.1

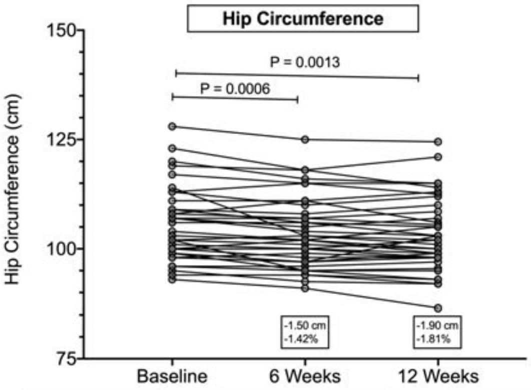

Uncorrected Fisher's LSD	Mean Diff.	95.00% CI of diff.	Significant?	Summary	Individual P Value
Baseline vs. 6 Weeks	1.463	0.6713 to 2.254	Yes	***	0.0006
Baseline vs. 12 Weeks	1.900	0.7908 to 3.009	Yes	**	0.0013

	Baseline	6 Weeks	12 Weeks
Mean	105.0	103.5	103.1

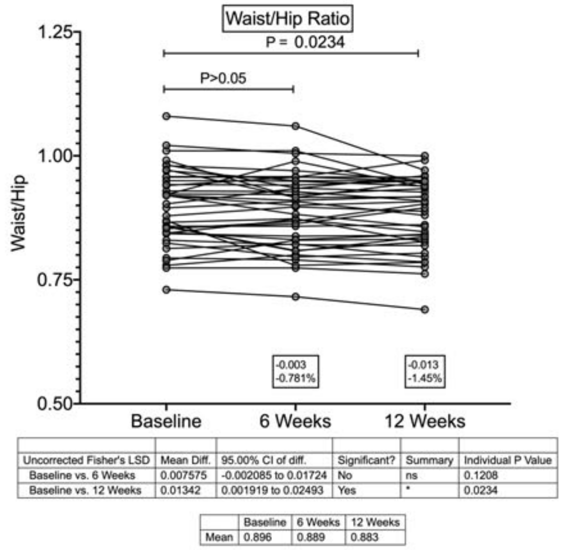

Uncorrected Fisher's LSD	Mean Diff.	95.00% CI of diff.	Significant?	Summary	Individual P Value
Baseline vs. 6 Weeks	0.007575	-0.002085 to 0.01724	No	ns	0.1208
Baseline vs. 12 Weeks	0.01342	0.001919 to 0.02493	Yes	*	0.0234

	Baseline	6 Weeks	12 Weeks
Mean	0.896	0.889	0.883

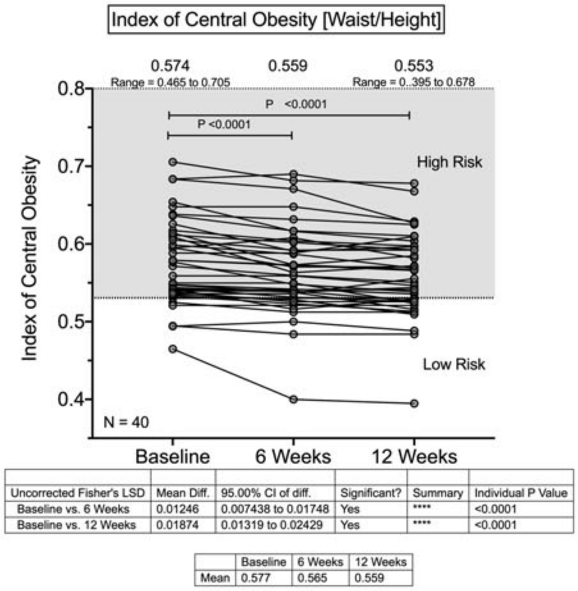

Uncorrected Fisher's LSD	Mean Diff.	95.00% CI of diff.	Significant?	Summary	Individual P Value
Baseline vs. 6 Weeks	0.01246	0.007438 to 0.01748	Yes	****	<0.0001
Baseline vs. 12 Weeks	0.01874	0.01319 to 0.02429	Yes	****	<0.0001

	Baseline	6 Weeks	12 Weeks
Mean	0.577	0.565	0.559

Shaded areas represent normal, reference range

SUPPLEMENTARY FIG. S1. Individual subject anthropometric values at baseline and completion of weeks 6 and 12.
