# Supplementary material for: Nigella sativa and Trigonella foenum-graecum Supplemented Chapatis Safely Improve HbA1c, Body Weight, Waist Circumference, Blood Lipids, and Fatty Liver in Overweight and Diabetic Subjects: A Twelve-Week Safety and Efficacy Study
Source: J Med Food. 2020 Sep 2;23(9):905–19. doi: 10.1089/jmf.2020.0075 (PMC7478223; doi:10.1089/jmf.2020.0075)
Supplement: Supplemental data [file Supp_Fig2.pdf]

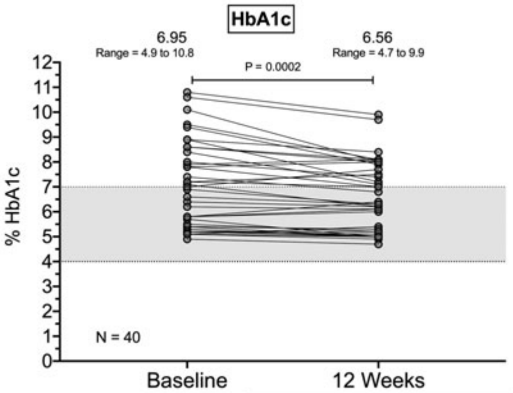

	Baseline	12 Weeks
Mean	6.95	6.56
Std. Deviation	1.72	1.40
Std. Error of Mean	0.272	0.221
Lower 95% CI of mean	6.40	6.11
Upper 95% CI of mean	7.50	7.01

Paired t test	
P value	0.0002
P value summary	***
Significantly different (P < 0.05)?	Yes
One- or two-tailed P value?	Two-tailed
t, df	t=4.08, df=39
Number of pairs	40

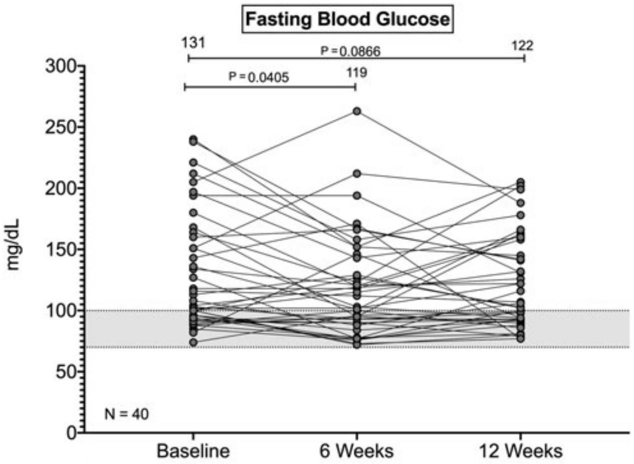

Uncorrected Fisher's LSD	Mean Diff.	95.00% CI of diff.	Significant?	Summary	Individual P Value
Baseline vs. 6 Weeks	12.2	0.550 to 23.7	Yes	*	0.0405
Baseline vs. 12 Weeks	9.10	-1.37 to 19.6	No	ns	0.0866

	Baseline	6 Weeks	12 Weeks
Mean	131	119	122

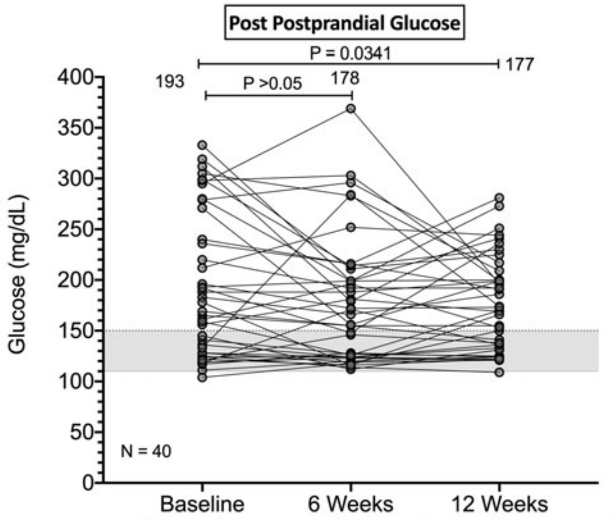

Uncorrected Fisher's LSD	Mean Diff.	95.00% CI of diff.	Significant?	Summary	Individual P Value
Baseline vs. 6 Weeks	14.4	-2.94 to 31.6	No	ns	0.1012
Baseline vs. 12 Weeks	16.2	1.28 to 31.2	Yes	*	0.0341

	Baseline	6 Weeks	12 Weeks
Mean	193	178	177

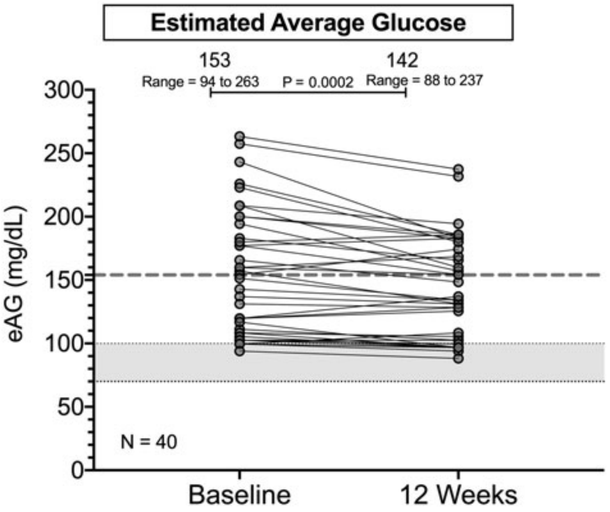

Paired t test	
P value	0.0002
P value summary	***
Significantly different (P < 0.05)?	Yes
One- or two-tailed P value?	Two-tailed
t, df	t=4.08, df=39
Number of pairs	40

SUPPLEMENTARY FIG. S2. Individual subject glycemic values at baseline and completion of weeks 6 and 12.
