# Supplementary material for: Nigella sativa and Trigonella foenum-graecum Supplemented Chapatis Safely Improve HbA1c, Body Weight, Waist Circumference, Blood Lipids, and Fatty Liver in Overweight and Diabetic Subjects: A Twelve-Week Safety and Efficacy Study
Source: J Med Food. 2020 Sep 2;23(9):905–19. doi: 10.1089/jmf.2020.0075 (PMC7478223; doi:10.1089/jmf.2020.0075)
Supplement: Supplemental data [file Supp_Fig3.pdf]

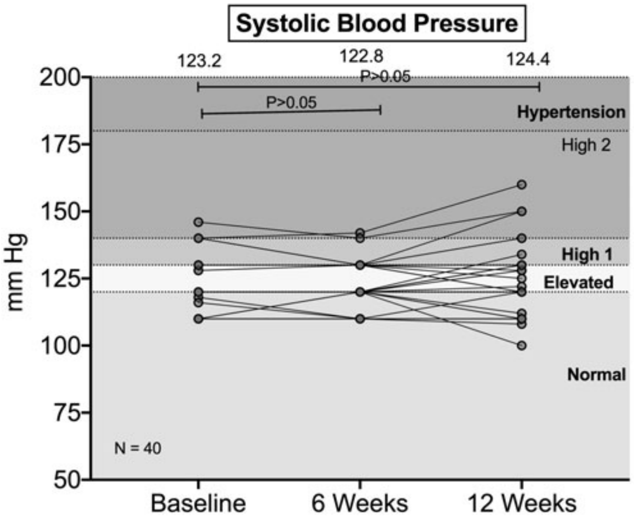

Uncorrected Fisher's LSD	Mean Diff.	95.00% CI of diff.	Significant?
Baseline vs. 6 Weeks	0.350	-1.05 to 1.75	No
Baseline vs. 12 Weeks	-1.22	-4.08 to 1.63	No

Repeated measures ANOVA summary	
Assume sphericity?	No
F	0.941
P value	0.3654
P value summary	ns
Statistically significant (P < 0.05)?	No
Geisser-Greenhouse's epsilon	0.688
R squared	0.0236

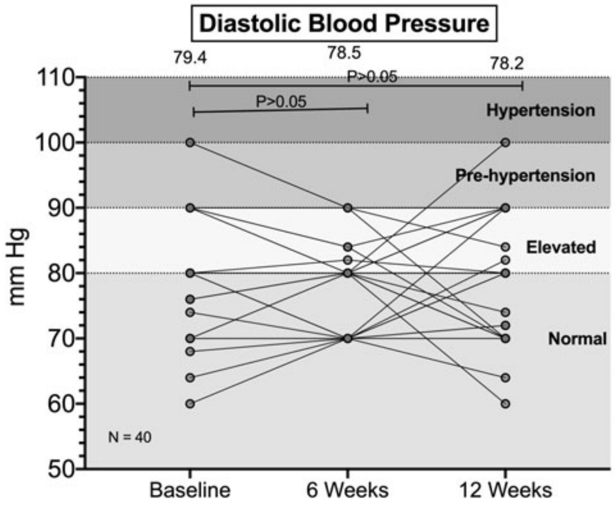

Uncorrected Fisher's LSD	Mean Diff.	95.00% CI of diff.	Significant?
Baseline vs. 6 Weeks	0.850	-0.887 to 2.59	No
Baseline vs. 12 Weeks	1.15	-2.37 to 4.67	No

Repeated measures ANOVA summary	
Assume sphericity?	No
F	0.3477
P value	0.6286
P value summary	ns
Statistically significant (P < 0.05)?	No
Geisser-Greenhouse's epsilon	0.6923
R squared	0.008837

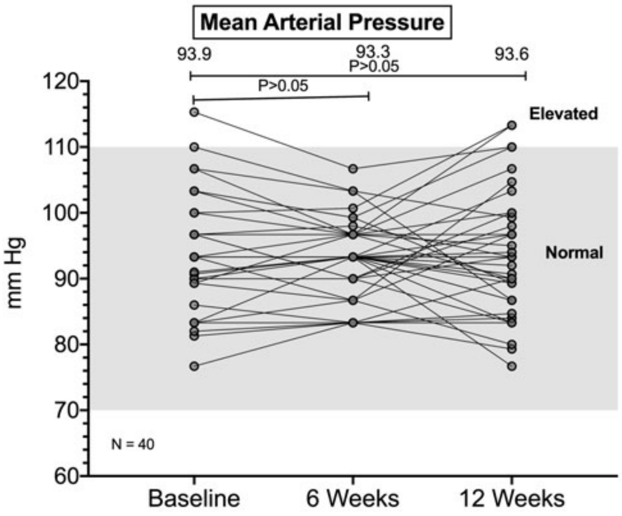

Uncorrected Fisher's LSD	Mean Diff.	95.00% CI of diff.	Significant?
Baseline vs. 6 Weeks	0.850	-0.887 to 2.59	No
Baseline vs. 12 Weeks	1.15	-2.37 to 4.67	No

	Baseline	6 Weeks	12 Weeks
Mean	93.9	93.3	93.6
Std. Deviation	8.05	5.74	9.18
Std. Error of Mean	1.27	0.907	1.45
Lower 95% CI of mean	91.4	91.4	90.7
Upper 95% CI of mean	96.5	95.1	96.5

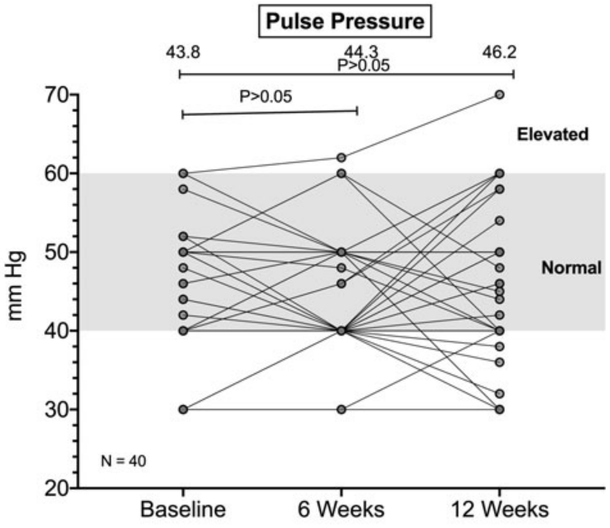

Uncorrected Fisher's LSD	Mean Diff.	95.00% CI of diff.	Significant?
Baseline vs. 6 Weeks	-0.5000	-3.569 to 2.569	No
Baseline vs. 12 Weeks	-2.375	-5.444 to 0.6940	No

Repeated measures ANOVA summary	
Assume sphericity?	Yes
F	1.319
P value	0.2732
P value summary	ns
Statistically significant (P < 0.05)?	No

SUPPLEMENTARY FIG. S3. Individual subject blood pressure values at baseline and completion of weeks 6 and 12.
