# Supplementary figures and images for: Nigella sativa and Trigonella foenum-graecum Supplemented Chapatis Safely Improve HbA1c, Body Weight, Waist Circumference, Blood Lipids, and Fatty Liver in Overweight and Diabetic Subjects: A Twelve-Week Safety and Efficacy Study
Source: J Med Food. 2020 Sep 2;23(9):905–19. doi: 10.1089/jmf.2020.0075 (PMC7478223; doi:10.1089/jmf.2020.0075)

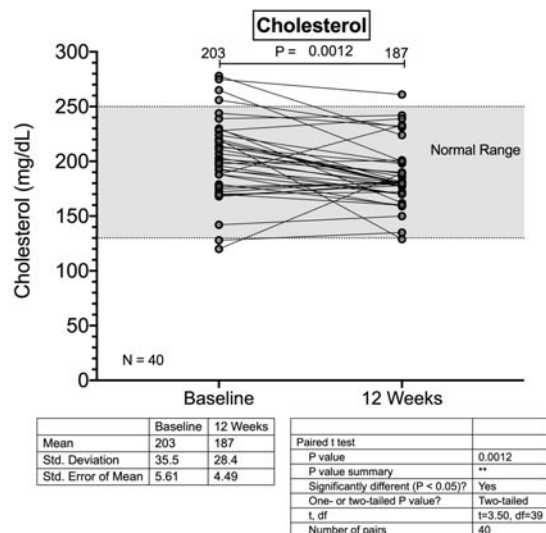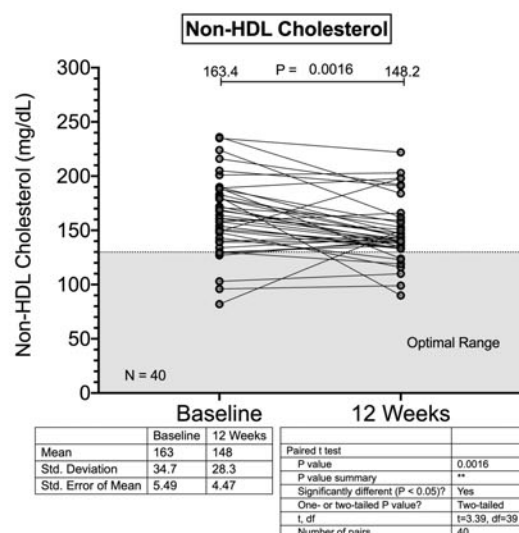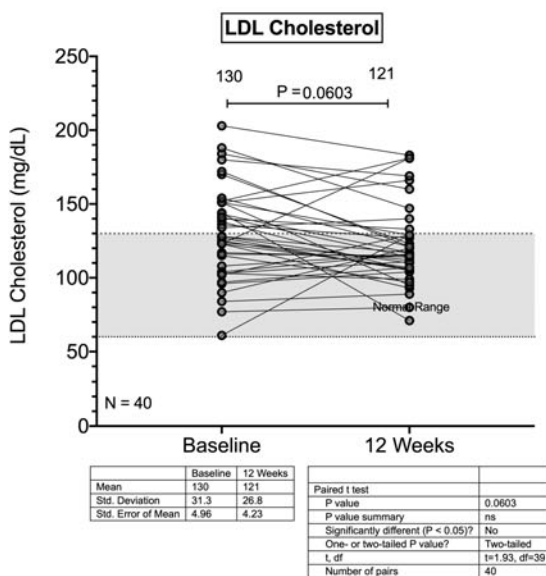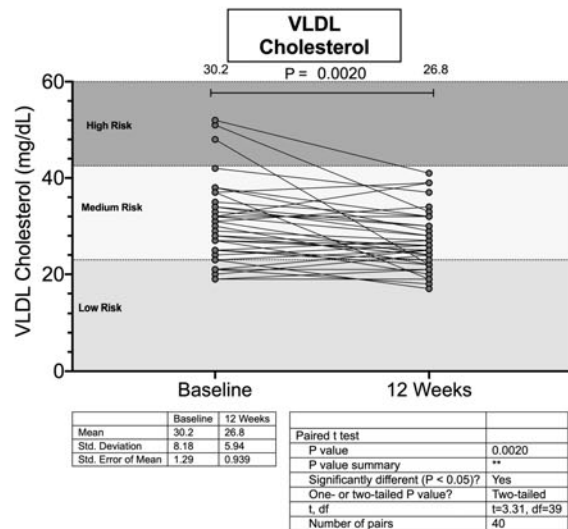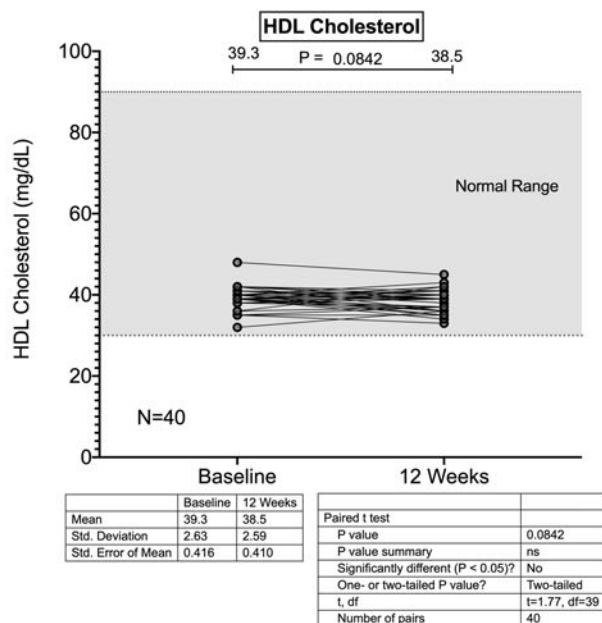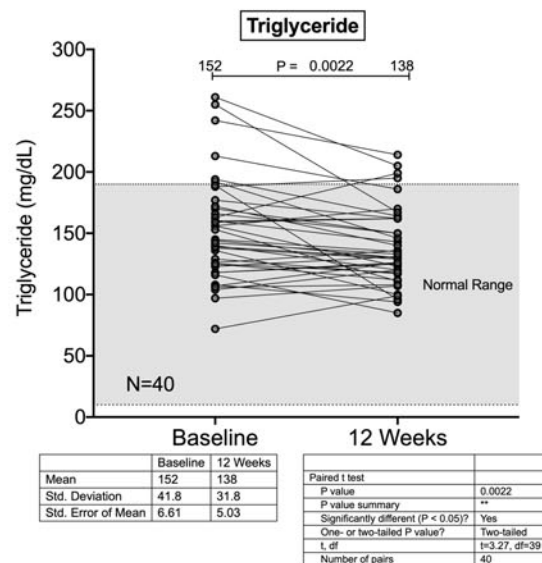

**SUPPLEMENTARY FIG. S4.** Individual subject lipidic profiles at baseline and completion of week 12.

Supplement: Supplemental data [file Supp_Fig4.pdf]

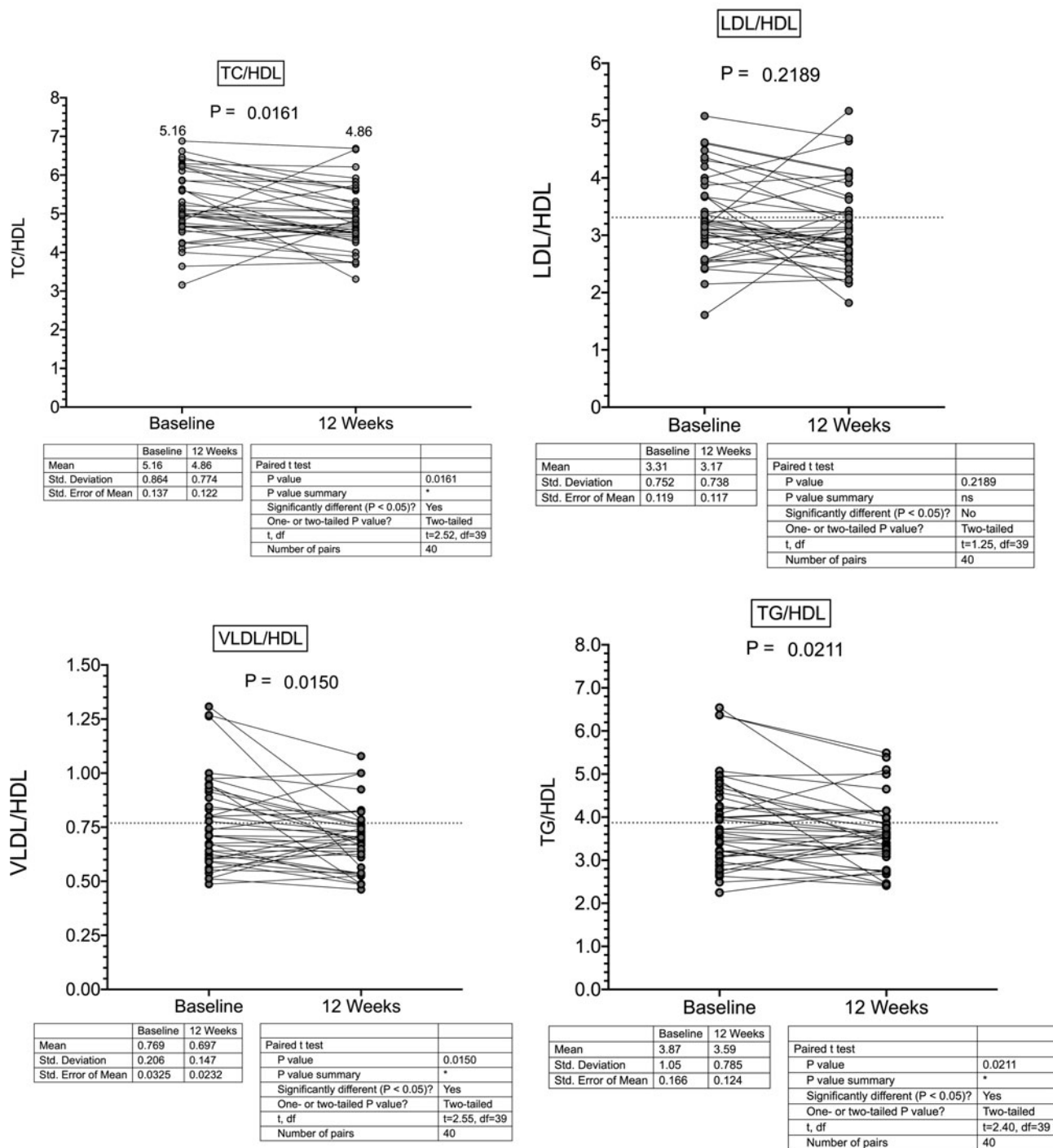

**SUPPLEMENTARY FIG. S5.** Individual subject lipid ratios at baseline and completion of week 12.

Supplement: Supplemental data [file Supp_Fig5.pdf]
