# Supplementary material for: Nigella sativa and Trigonella foenum-graecum Supplemented Chapatis Safely Improve HbA1c, Body Weight, Waist Circumference, Blood Lipids, and Fatty Liver in Overweight and Diabetic Subjects: A Twelve-Week Safety and Efficacy Study
Source: J Med Food. 2020 Sep 2;23(9):905–19. doi: 10.1089/jmf.2020.0075 (PMC7478223; doi:10.1089/jmf.2020.0075)
Supplement: Supplemental data [file Supp_Fig7.pdf]

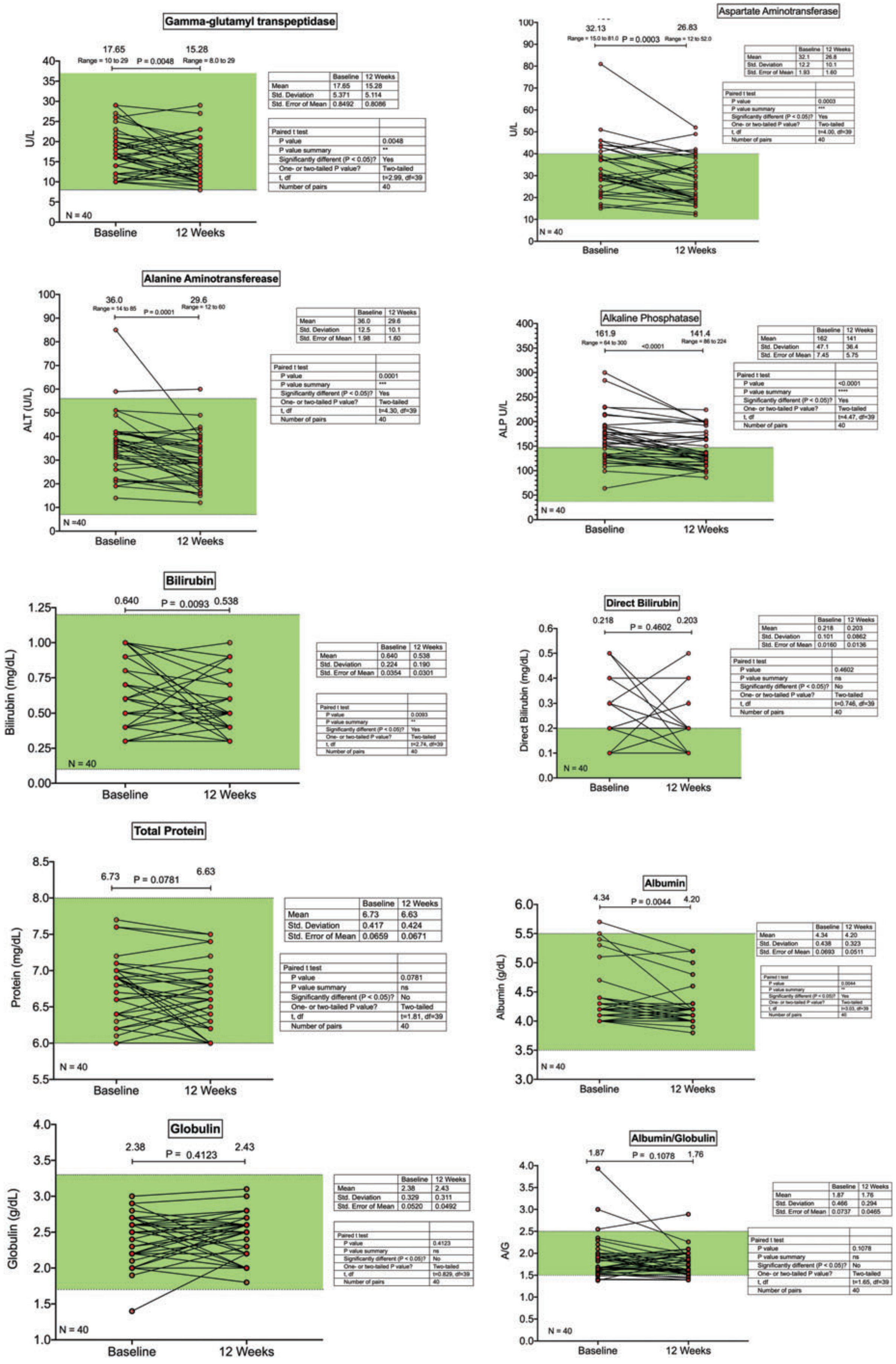

SUPPLEMENTARY FIG. S7. Individual subject kidney and thyroid profiles at baseline and completion of week 12.
